# Biosynthesis of methyl-proline containing griselimycins, natural products with anti-tuberculosis activity†
†Electronic supplementary information (ESI) available. See DOI: 10.1039/c7sc02622f



**DOI:** 10.1039/c7sc02622f

**Published:** 2017-08-03

**Authors:** Peer Lukat, Yohei Katsuyama, Silke Wenzel, Tina Binz, Claudia König, Wulf Blankenfeldt, Mark Brönstrup, Rolf Müller

**Affiliations:** a Helmholtz Institute for Pharmaceutical Research Saarland (HIPS) , Helmholtz Center for Infection Research and Pharmaceutical Biotechnology , Saarland University Campus , Building C2.3 , 66123 Saarbrücken , Germany . Email: Rolf.Mueller@helmholtz-hzi.de; b Structure and Function of Proteins , Helmholtz Centre for Infection Research , Inhoffenstr. 7 , 38124 Braunschweig , Germany; c Sanofi Aventis Deutschland , Industriepark Höchst , 65926 Frankfurt , Germany; d Institute of Biochemistry, Biotechnology and Bioinformatics , Technische Universität Braunschweig , Spielmannstr. 7 , 38106 Braunschweig , Germany; e Department for Chemical Biology , Helmholtz Centre for Infection Research , Inhoffenstr. 7 , 38124 Braunschweig , Germany; f German Centre for Infection Research Association (DZIF) , Partner site Hannover–Braunschweig , Germany

## Abstract

Griselimycins (GMs) are depsidecapeptides with superb anti-tuberculosis activity. They contain up to three (2*S*,4*R*)-4-methyl-prolines (4-MePro), of which one blocks oxidative degradation and increases metabolic stability in animal models. The natural congener with this substitution is only a minor component in fermentation cultures. We showed that this product can be significantly increased by feeding the reaction with 4-MePro and we investigated the molecular basis of 4-MePro biosynthesis and incorporation. We identified the GM biosynthetic gene cluster as encoding a nonribosomal peptide synthetase and a sub-operon for 4-MePro formation. Using heterologous expression, gene inactivation, and *in vitro* experiments, we showed that 4-MePro is generated by leucine hydroxylation, oxidation to an aldehyde, and ring closure with subsequent reduction. The crystal structures of the leucine hydroxylase GriE have been determined in complex with substrates and products, providing insight into the stereospecificity of the reaction.

## Abstract 2

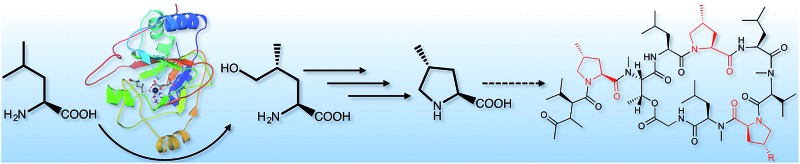
The biosynthesis of griselimycins in *Streptomyces* DSM 40835 and the pathway that stereospecifically converts l-leucine to (2*S*,4*R*)-4-methyl-proline are reported by means of biochemical and structural analysis.

## Introduction

Tuberculosis (TB) remains a major global health burden with an estimated 1.5 million deaths and 9.6 million new cases in 2014. Increased multidrug-resistance (3.3% of new cases, more than 30% in some countries)^1^ makes the development of new drugs against TB-causing *Mycobacterium tuberculosis* an urgent need.

**Fig. 1:**
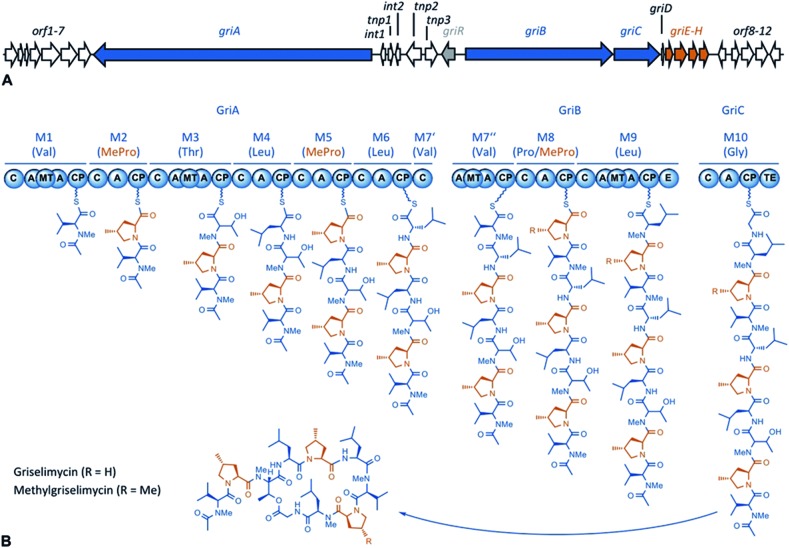
A GM biosynthetic gene cluster and an NRPS assembly line. (A) The GM biosynthetic gene cluster. The NRPS modules are shown in blue, and the genes associated with (2*S*,4*R*)-4-MePro biosynthesis are shown in red. (Annotation: Table S1†) (B) The GM assembly line. The NRPS modules are numbered 1–10, and the substrates are given in parentheses. For each module, the domains are: C = condensation, A = adenylation, MT = methyl transferase, CP = carrier protein, E = epimerase, and TE = thioesterase. (2*S*,4*R*)-4-MePro is shown in orange.

Griselimycins (GMs), cyclic depsidecapeptides (Fig. 1) isolated from the *Streptomyces* strain DSM 40835, were reported as effective against drug-resistant *M. tuberculosis* in the 1960s, but their development was abandoned due to their poor pharmacokinetic properties.^2–6^ In a re-assessment of GMs, we have recently shown that GMs target DnaN, the sliding clamp of DNA-polymerase, thereby circumventing common forms of TB drug resistance.^7^ To improve the *in vivo* properties of GMs, we searched for metabolically labile sites and found that degradation starting with the oxidation of proline at position 8 of the GM was a major issue.^7^ This agrees with the observation that synthetic GMs carrying a cyclohexyl moiety at Pro8 and the natural derivative methyl-GM (MGM), which incorporates the non-proteinogenic amino acid (2*S*,4*R*)-4-methylproline ((2*S*,4*R*)-4-MePro) at this position, were significantly more stable upon incubation with human liver microsomes.^7^ Other GMs also incorporate (2*S*,4*R*)-4-MePro at positions 2 and 5, but MGMs are formed in far lower amounts than GMs.

To understand (2*S*,4*R*)-4-MePro biosynthesis and set the stage to enhance MGM yields systematically, we unravelled the generation of (2*S*,4*R*)-4-MePro within GM biosynthesis and studied the factors controlling the incorporation of amino acids into GMs.

## Results & discussion

Using sequencing, retrobiosynthetic analysis and inactivation experiments, we identified the GM biosynthetic gene cluster of *Streptomyces* DSM 40835.^7,8^ The cluster contains 26 open reading frames, including three non-ribosomal peptide synthetases (NRPSs) with six, three and one modules, which build the decapeptide architecture of GMs (Table S1†). Based on antiSMASH 3.0 gene cluster analysis,^9^ the adenylation (A) domain substrate specificities of the ten NRPS modules were correctly predicted for positions 3, 8 and 10 (Thr3, Pro8 and Gly10; Table S2†). For A domains 2, 5 and 8, proline incorporation was predicted, showing that the applied method does not discriminate between Pro and 4-MePro. For leucine-incorporating modules 4, 6 and 9, the substrate predictions were incongruent and the valine-incorporating modules 1 and 7 were assigned to threonine instead. Despite these limitations, the GM assembly line could be defined as shown in Fig. 1.

**Fig. 2:**
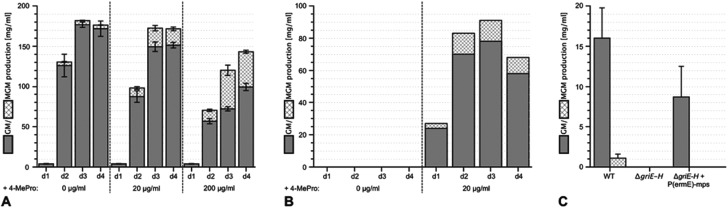
Enhancing the yield of methyl-griselimycin by feeding with (2*S*,4*R*)-4-methyl-proline and the complementation of the Δ*griE*–*H* mutant. (A) Liquid cultures of the wild-type griselimycin producer strain *Streptomyces* DSM 40835 were supplemented with 0, 20 or 200 μg ml^–1^ (2*S*,4*R*)-4-methyl-proline. Samples were taken after 1, 2, 3 and 4 days of incubation. Their griselimycin and methyl-griselimycin content was determined by UHPLC (Ultra High Performance Liquid Chromatography). (B) The analysis of (methyl-)griselimycin production in the Δ*griE*–*H* mutant strain with and without an external supply of 20 μg ml^–1^ (2*S*,4*R*)-4-methyl-proline to the cultivation medium. Samples were taken after 1, 2, 3 and 4 days of incubation. (C) Analysis of (methyl-)griselimycin production after genetic complementation of the Δ*griE*–*H* mutant by chromosomal integration of the *griE*–*H* expression construct P(ermE)-mps. Analysis was carried out for the comparison of the wild-type griselimycin producer (WT) and the deletion mutant strain.

To study 4-MePro-incorporation further, the specificities of the recombinantly produced domains A2, A5 and A8 were investigated (Fig. S1†). While they preferred (2*S*,4*R*)-4-MePro, the relative activity of A8 towards proline was 1.5 fold higher than in the case of A2 and 2.0 fold higher compared to that of A5. We speculated that the cellular 4-MePro availability might be a limitation for MGM formation, and thus performed feeding experiments employing synthetic 4-MePro. The results further proved the substrate tolerance of the A8 domain as the MGM yield could be increased from less than 3% to more than 30% of the total GM/MGM production by adding 0.2 grams of (2*S*,4*R*)-MePro per litre to shaking cultures of the producer strain (Fig. 2A, Table S3†).

To understand the biosynthesis of (2*S*,4*R*)-MePro in *Streptomyces* DSM 40835, we aimed to reconstitute its biosynthesis *in vitro* and *in vivo*. 4-MePro incorporation has been shown for several natural products (Fig. S2†), and its biosynthesis has been investigated in the context of nostopeptolide generation by *Nostoc* cyanobacteria,^10,11^ yielding the (2*S*,4*S*)-diastereomer, and of echinocandins by the fungus *Emericella rugulosa*,^12^ giving (2*S*,4*R*)-4-MePro as in the GMs (Fig. S3†). Both of these pathways start with the hydroxylation of l-leucine at position five by a Fe(ii)/α-ketoglutarate-dependent hydroxylase. While the cyanobacterial enzyme LdoA produces (2*S*,4*S*)-5-hydroxyleucine, the fungal EcdK produces the (2*S*,4*R*)-diastereomer, thereby defining the stereochemistry at C4 of the final product. Further oxidation generates 4-methylglutamate-5-semialdehyde, either *via* a second EcdK-catalyzed hydroxylation to the aldehyde hydrate or by the dehydrogenase NosE. Spontaneous cyclization to 3-methyl-Δ^1^-pyrroline-5-carboxylic acid followed by reduction then furnishes 4-MePro (Fig. S3†).

Sequence analysis identified *griE*, *griF* and *griH* as encoding for enzymes that might catalyze similar biochemistry. Like LdoA and EcdK, GriE belongs to the 2OG-Fe(ii) oxygenase superfamily, whose members couple the decarboxylation of α-ketoglutarate to substrate oxygenation/hydroxylation *via* an oxo-ferryl intermediate. Despite low sequence identity (approx. 13%; Fig. S4A†), we speculated that GriE may initiate 4-MePro biosynthesis from l-leucine in a similar manner as in nostopeptolide or echinocandin biosynthesis. GriF is a zinc-dependent dehydrogenase that shares 34% sequence identity with NosE from the 4-MePro biosynthetic pathway of *N. punctiforme* (Fig. S4B†), indicating that it may convert 4-hydroxyleucine to 4-methylglutamate-5-semialdehyde. GriG is a type II thioesterase (Fig. S4C†) and, as such, is not expected to be involved in 4-MePro generation. GriH was annotated as an F420-dependent oxidoreductase, sharing high sequence identity with several enzymes proposed to be involved in pyrroline reduction towards propylproline generation (Fig. S4D†), similar to the unrelated NosF in cyanobacterial 4-MePro (Fig. S3A†)^10^ and ProC in the proline biosynthesis of *E. coli.* ^13^

The hypothesis that 4-MePro biosynthesis in *Streptomyces* DSM 40835 follows a similar route was reinforced by feeding experiments employing deuterated l-leucine, which unambiguously showed incorporation into all 4-MePro units (Fig. S5†). Further, a GM/MGM-deficient *griE*–*H* knock out mutant was generated (ESI II.1.3†) and supplemented with (2*S*,4*R*)-4-MePro or complemented with *griE*–*H* under the control of a constitutive promoter. In both cases, GM/MGM production was restored (Fig. 2B and C), demonstrating that the genes required for 4-MePro formation are indeed found in this sub-operon of the *gri*-cluster. The expression of *griE*–*H* in *S. lividans* induced the production of (2*S*,4*R*)-4-MePro (Fig. S6†), whereas the deletion of *griE* or *griF* abolished it. 5-Hydroxyleucine accumulated in the Δ*griF* mutant, indicating that GriE indeed produces 5-hydroxyleucine as the first step in 4-MePro biosynthesis. The deletion of *griH* did not have an effect, suggesting that its potential activity as a terminal oxidoreductase may be complemented by *proC* from proline biosynthesis.

**Fig. 3:**
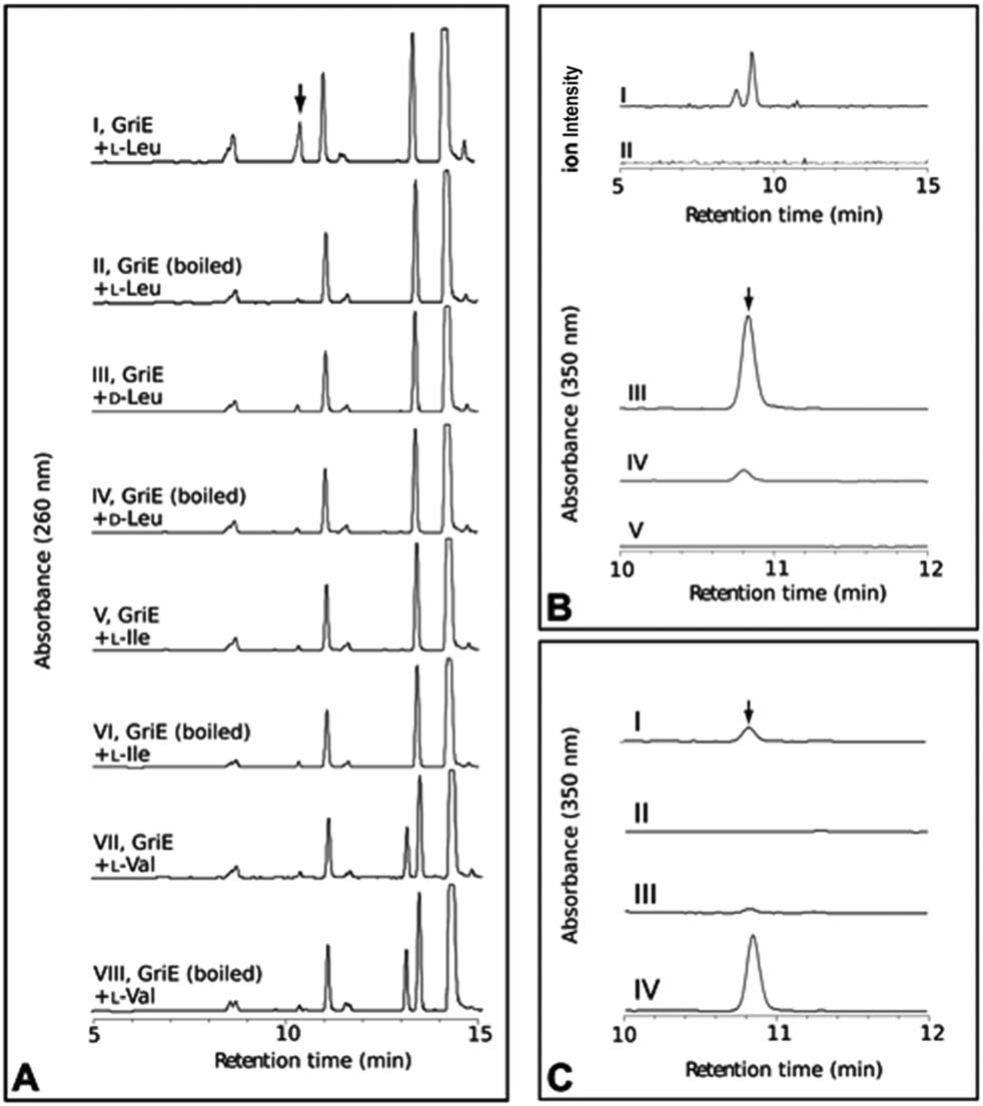
*In vitro* analysis of the purified proteins GriE, GriF and GriH. (A) GriE: (I) 5-hydroxyleucine was detected by LC-MS analysis after the incubation of GriE with l-leucine and α-ketoglutarate. (II) Heat inactivated GriE did not yield any 5-hydroxyleucine. 5-Hydroxyleucine was characterized as described in the ESI, Sections 3.10 and 3.11.† No reaction products were obtained using d-leucine, l-isoleucine or l-valine as substrates (III–VIII). (B) The extracted ion chromatogram (*m*/*z* 128) corresponding to (3*R*,5*S*)-3-methyl-Δ^1^-pyrroline-5-carboxylic acid in positive ion mode (I and II). GriF: (I) the formation of (3*R*,5*S*)-3-methyl-Δ^1^-pyrroline-5-carboxylic acid was observed upon the incubation of GriF with NAD^+^ and 5-hydroxyleucine. (II) Heat inactivated GriF did not yield any (3*R*,5*S*)-3-methyl-Δ^1^-pyrroline-5-carboxylic acid. (III) The incubation of GriF with ProC from *E. coli*, 5-hydroxyleucine and NAD^+^ resulted in the formation of (2*S*,4*R*)-4-methyl-proline. (IV) The reaction of GriF in the absence of ProC resulted in trace amounts of (2*S*,4*R*)-4-methyl-proline, probably due to a nonenzymatic reduction due to the presence of DTT as a reductant. (V) No formation of (2*S*,4*R*)-4-methyl-proline could be detected in the absence of GriF. (C) GriH coupled to GriF: (I) the formation of (2*S*,4*R*)-4-MePro was observed upon the incubation of the enzymes with NADH, NAD^+^, 5-hydroxyleucine and cell extract of *S. lividans*. (II) Removal of GriF abolished the formation of (2*S*,4*R*)-4-MePro. (III) Removal of GriH lead to only trace amounts of (2*S*,4*R*)-4-MePro. (IV) The removal of cellular extract from the reaction resulted in higher amounts of (2*S*,4*R*)-4-MePro. The stereochemistry of the produced (2*S*,4*R*)-4-MePro was confirmed by Advanced Marfey’s Method with d-FDLA derivatized samples.

To elucidate the individual functions further, recombinant GriE and GriF were produced in *E. coli*, while *S. lividans* was required for the production of soluble GriH. The formation of 5-hydroxyleucine was observed upon the incubation of l-leucine with purified GriE, α-ketoglutarate, ascorbate, and FeSO_4_ (Fig. 3A). Purified 5-hydroxyleucine from *E. coli* overexpressing *griE* was analyzed by ^1^H-NMR spectroscopy and compared with previously reported data,^10^ demonstrating that it is most likely (2*S*,4*R*)-5-hydroxyleucine or its (2*R*,4*S*)-diastereomer. No turnover was detected with d-leucine, l-valine or l-isoleucine, pointing to the strict substrate specificity of GriE. Double-hydroxylation to 5,5-dihydroxyleucine, as reported for EcdK produced by echinocandin biosynthesis,^12^ was not observed.

The product of the incubation of recombinant GriF with 5-hydroxyleucine, ZnSO_4_ and NAD^+^ was unstable and yields were too low for purification and NMR analysis, but LC-MS analysis of the supernatant showed a mass (*m*/*z* 128) corresponding to the expected species 3-methyl-Δ^1^-pyrroline-5-carboxylic acid (Fig. 3B I). Thus, we included ProC, the enzyme that catalyses the final reduction in proline biosynthesis in *E. coli*, hypothesizing that ProC could also reduce this intermediate to generate 4-MePro. This was indeed confirmed (Fig. 3B III).

**Fig. 4:**
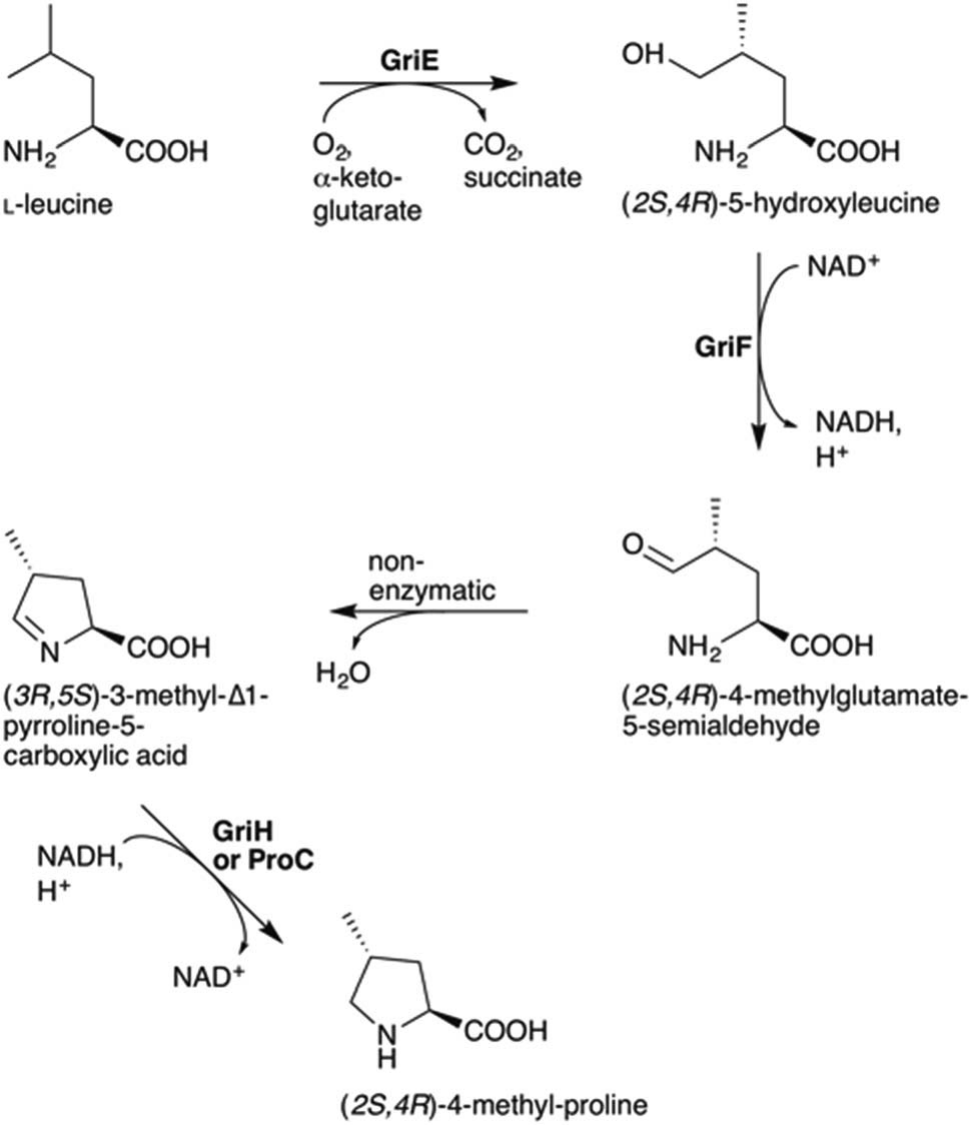
Proposed pathway for (2*S*,4*R*)-4-MePro formation in griselimycin biosynthesis.

Recombinant GriH purified from *S. lividans* TK24 lacked the characteristic fluorescence of the expected F420 co-factor but nevertheless led to 4-MePro in a coupled assay with GriF, indicating that GriH can use NADH directly without requiring F420 (Fig. 3C). Together, these experiments suggest that 4-MePro biosynthesis starts with the GriE-mediated hydroxylation of l-leucine, followed by oxidation with GriF. Spontaneous cyclization and reduction by GriH then give 4-MePro for MGM biosynthesis (Fig. 4).

The proposed pathway is similar to the one of *Nostoc* with the key difference being the inverted chiral center at C4 of 4-MePro as a consequence of the initial GriE-catalyzed step. For corroboration and to understand the stereoselectivity of this hydroxylation, we determined the crystal structures of GriE in the ligand-free state and from crystals formed in the presence of l-leucine and α-ketoglutarate. Fe^2+^ was replaced by the less oxidation-sensitive Co^2+^ or Mn^2+^, yielding crystals that diffracted to 1.82 Å for the apo form, 1.76 Å for the Co^2+^- and 1.53 Å for the Mn^2+^-ligand complex. The models were refined to *R*/*R*_free_ values of 17.2/21.3% (apo, PDB: ; 5NCH), 14.0/16.9% (Co^2+^, PDB: ; 5NCI) and 15.5/17.3% (Mn^2+^, PDB: ; 5NCJ) (Table S4†).

**Fig. 5:**
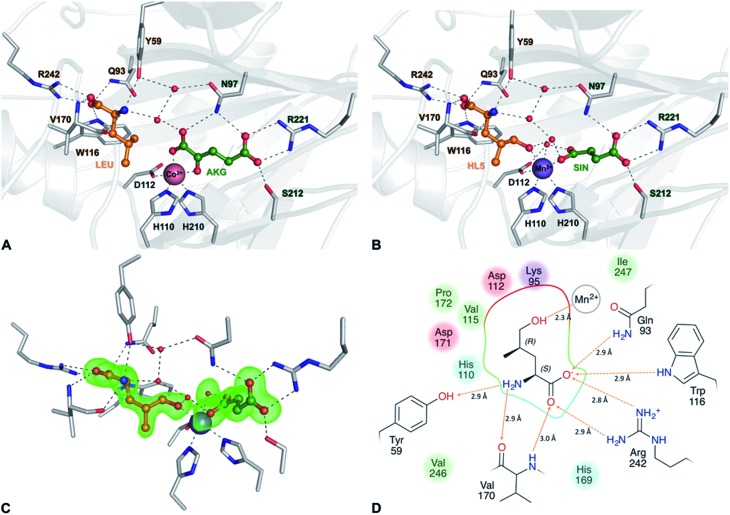
Crystal structures of the GriE substrate and product complexes. (A) Structure of the enzyme co-crystallized with Co^2+^, l-leucine (LEU, orange) and α-ketoglutarate (AKG, green). (B) Active site of the Mn^2+^-containing enzyme with the reaction products (2*S*,4*R*)-5-hydroxyleucine (HL5, orange) and succinate (SIN, green). (C) |*F*_O_ *F*_C_| difference electron density of 5-hydroxyleucine and succinate, contoured at 3*σ*. (D) Ligand interaction diagram for the reaction product (2*S*,4*R*)-5-hydroxyleucine. Hydrogen bonds are shown as orange arrows, dashed for side chain-ligand bonds and solid for main chain-ligand bonds. Metal coordination is shown as an orange line. The binding pocket and residues forming it are highlighted according to their physicochemical properties (green: hydrophobic, blue: polar, red: negatively charged, and purple: positively charged).

**Fig. 6:**
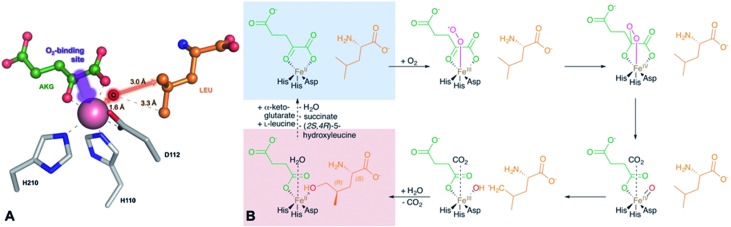
Implications of the GriE crystal structures on the reaction mechanism. (A) Active site as observed in the crystal structure of the substrate complex. O_2_ would bind to the vacant coordination site (purple arrow). After the decarboxylation of α-ketoglutarate, the reactive oxygen intermediate (O) would abstract a hydrogen atom from the nearest methyl group of l-leucine (red arrow), leading to (2*S*,4*R*)-5-hydroxyleucine (see also Fig. S9 and S10†). (B) Reaction scheme derived from the crystal structures of the GriE ligand complexes. Starting with the state corresponding to the Co^2+^/substrate-containing crystal structure (highlighted in blue), dioxygen can bind to the vacant coordination site *trans* to His110 and perform a nucleophilic attack on the carbonyl group of α-ketoglutarate, forming a Fe(iv)-peroxy-hemiketal transition state. In order to react with the substrate, the reactive oxygen species has to swap its position with CO_2_ during the subsequent decarboxylation step. Alternatively, CO_2_ might already have left the active site and been replaced by a water molecule (not shown). The oxo-ferryl intermediate abstracts a hydrogen from the closest C_δ_ methyl group of l-leucine, leaving a radical at the substrate which then reacts with the hydroxyl group of the Fe(iii)-hydroxo species. The state with the reaction products succinate and (2*S*,4*R*)-5-hydroxyleucine still bound to the metal, but CO_2_ being replaced by a water molecule, corresponds to the crystal structure of the Mn^2+^/product complex (highlighted in red).

GriE possesses the typical fold of other Fe(ii)/α-ketoglutarate dependent dioxygenases (Fig. S7†). The canonical iron-binding motif is formed by His110, Asp112 and His210 and has lost the metal in the apo structure. The crystal structure obtained by co-crystallization with Co^2+^ and substrates unambiguously revealed bound metal and both l-leucine and α-ketoglutarate (Fig. S8A and B†). Co^2+^ is coordinated by the iron-binding motif and by α-ketoglutarate as a bi-dentate ligand through its C-1 carboxylate and C-2 keto group (Fig. 5A, 6A and S9†). The axial position *trans* to His110, the expected binding site of oxygen, remains vacant. l-Leucine does not coordinate to the metal ion but is tightly bound *via* its carboxy and amino groups. Its side chain is not involved in the interactions and adopts a conformation that corresponds to the most probable rotamer (Fig. S10†).

To our surprise, difference electron density in the complex obtained with Mn^2+^, l-leucine and α-ketoglutarate corresponded to the products 5-hydroxyleucine and succinate, indicating turnover despite the Fe^2+^/Mn^2+^ exchange (Fig. 5C, S8C and D†). The well-defined electron density clearly confirms 5-hydroxyleucine to be the postulated (2*S*,4*R*)-diastereomer, as required for the stereospecific generation of (2*S*,4*R*)-4-MePro in the pathway shown in Fig. 4. The position of the reaction products is similar to the substrate complex, but succinate coordinates the metal with one carboxylate oxygen occupying the position of the α-keto group of α-ketoglutarate in the substrate complex, while the previously empty oxygen binding site is occupied by a water molecule. The hydroxyl group of (2*S*,4*R*)-5-hydroxyleucine takes the position of one of the metal-coordinating carboxylate oxygen atoms of α-ketoglutarate, establishing a tight interaction with the cation (Fig. 5B, D and S9†).

Superposition of the three crystal structures showed no conformational differences between the substrate and product complexes, while conformational changes in the three loop regions compared to the apo form were visible (Fig. S11A†): the loop comprising residues 48–57 is shifted towards the active site in the ligand-containing structures and thus seems to be involved in the closing of the active site after substrate binding. While the residues 159–176 were not visible in the electron density of the apo structure, this region is well defined in the ligand complexes. As Val170 is involved in hydrogen bonding to 5-hydroxy-leucine *via* its peptide backbone, it can be assumed that this loop functions as a lid for the active site and, by closing on substrate binding, prevents the release of harmful reactive oxygen species during the reaction. A third loop comprising residues 232–247 is tilted away from the active site in the ligand complexes. This is linked to Arg242, which is pushed outwards when forming a salt-bridge to the carboxylate of 5-hydroxy-leucine. These observations are supported by slightly elevated B-factors indicating the increased conformational flexibility of these loops (Fig. S11B–D†).

Fe(ii)/α-ketoglutarate dependent enzymes are well studied^14^ and thus a reaction mechanism can be postulated in which both ligand-containing crystal structures correspond to distinct states within the reaction cycle of GriE (Fig. 6B and S9†): in the Co^2+^/substrate containing crystal structure, l-leucine is bound in the vicinity of the metal albeit without direct contacts, while α-ketoglutarate binds as a bi-dentate ligand. The coordination site *trans* to His110 remains vacant. This crystal structure should thus correspond to the substrate complex prior to the binding of dioxygen to the unoccupied coordination site of Fe(ii). Facilitated by the transfer of electron density from α-ketoglutarate to iron, dioxygen binds to the free coordination site in the next step. The activated dioxygen then performs a nucleophilic attack on the carbonyl group of α-ketoglutarate, forming a Fe(iv)-peroxy-hemiketal transition state that results in the subsequent decarboxylation of α-ketoglutarate. This generates an oxo-ferryl species (Fe(iv)), which will react with the substrate l-leucine. In order to perform the attack on the substrate, the reactive oxygen species has to rearrange to occupy a coordination site orthogonal to the initial dioxygen binding position and *trans* to His210. This rearrangement most likely occurs after the decarboxylation step. Although such a change of the oxygen coordination site during the reaction cycle is not common, it has been described in other members of this enzyme family, such as in anthocyanidin synthase,^15^ clavaminate synthase^16^ or cephalosporin synthase.^17,18^ The oxo-ferryl intermediate then abstracts a hydrogen atom from the nearest group of the substrate, leading to the formation of a radical that then reacts with the Fe(iii)-hydroxo species by abstraction of the hydroxyl group. In the GriE/substrate complex, both the C_δ_ methyl groups of l-leucine are located at a 4.4 Å distance to the metal. The reactive oxygen atom should be coordinated to the metal in a distance of approx. 1.6 Å.^19–21^ With respect to this position, the pro-S group would be closer to the reactive oxygen atom (3.0 Å) than the other C_δ_ methyl group (3.3 Å), explaining the exclusive formation of (2*S*,4*R*)-5-hydroxyleucine (Fig. 6A and S9†). The crystal structure of the Mn^2+^/product complex of GriE corresponds to this state with carbon dioxide having already dissociated from the metal and its coordination site being occupied by a water molecule. After the dissociation of succinate and (2*S*,4*R*)-5-hydroxyleucine, the reaction cycle is complete.

In order for the enzyme to produce the (2*S*,4*S*)-diastereomer l-leucine would have to adopt different rotamers. These rotamers of low probability would also cause steric clashes in the active centre (Fig. S10†), which makes the production of the (2*S*,4*S*)-diastereomer highly disfavoured and explains the exclusive formation of (2*S*,4*R*)-5-hydroxyleucine by GriE.

The (2*S*,4*S*)-5-hydroxyleucine-forming LdoA from *Nostoc punctiforme* and GriE belong to the same enzyme family but share only 14% sequence identity (Fig. S4A†). Although no crystal structure of LdoA is available, the superposition of GriE with a homology model of LdoA generated by Phyre2 ^22^ (Fig. S12†) shows that despite sharing the same jelly-roll core fold containing the iron-binding motif, the loops involved in substrate binding are not conserved. This suggests that the substrate in LdoA binds in a different orientation towards the active site, resulting in the formation of (2*S*,4*S*)-5-hydroxyleucine instead.

## Conclusions

We have described the biosynthesis of GM in *Streptomyces* DSM 40835 and investigated the basis for the incorporation of (2*S*,4*R*)-4-MePro. Biochemical and structural analysis unravelled the pathway that stereospecifically converts l-leucine to the (2*S*,4*R*)-diastereomer of 4-MePro. In addition to laying groundwork for improving GM-based anti-TB drugs (*e.g. via* feeding experiments), our work may also be useful for the biotechnological production of (2*S*,4*R*)-4-MePro in heterologous hosts using GriE and GriF together with the terminal enzyme of bacterial proline biosynthesis.
