# Supplementary material for: Biosynthesis of methyl-proline containing griselimycins, natural products with anti-tuberculosis activity
Source: Chem Sci. 2017 Aug 3;8(11):7521–7. doi: 10.1039/c7sc02622f (PMC5676206; doi:10.1039/c7sc02622f)
Supplement: Supplementary file 1 [file SC-008-C7SC02622F-s001.pdf]

Electronic Supplementary Material (ESI) for Chemical Science.
This journal is © The Royal Society of Chemistry 2017

Biosynthesis of methyl-proline containing griselimycins, natural products with anti-tuberculosis activity

Supplementary Information

Peer Lukat, Yohei Katsuyama, Silke Wenzel, Tina Binz, Claudia König, Wulf Blankenfeldt, Mark Brönstrup, Rolf Müller*

Contents

I. Materials **2**

II. Methods **2**

1. Identification and characterization of the griselimycin biosynthetic gene cluster 2
   1.1 Identification and decipherment of the entire griselimycin biosynthetic gene cluster sequence *2*
   1.2 In silico analysis of the griselimycin biosynthetic gene cluster sequence *4*
   1.3 Inactivation of the griselimycin biosynthesis pathway *4*
2. Substrate specificity of adenylation domains 5
   2.1 In silico analysis of adenylation domain substrate specificities *5*
   2.2 Biochemical analysis of adenylation domains A2, A5 and A8 .... *5*
   2.3 Feeding of (2*S*,4*R*)*-4-methyl-proline to* Streptomyces *DSM 40835* *6*
3. Methyl-proline biosynthesis 6
   3.1 Biosynthetic origin of methyl-proline *6*
   3.2 Complementation of the mps mutant to restore (methyl)griselimycin production *7*
   3.3 Heterologous expression of methyl-proline biosynthesis genes in S. lividans *TK24.* *8*
   3.4 Cloning, expression and purification of GriE *8*
   3.5 Cloning, expression and purification of GriF *9*
   3.6 Cloning, expression and purification of GriG *9*
   3.7 Cloning, expression and purification of GriH *10*
   3.8 Cloning, expression and purification of ProC (NC_010473.1, residues 343390 to 344199) from E. coli *10*
   3.9 In vitro analysis of GriE *11*
   3.10 Production of hydroxyleucine in BL21 (DE3) harboring pET28-GriE *11*
   3.11 NMR data of 5-hydroxyleucine *11*
   3.12 In vitro analysis of GriF *12*
   3.13 In vitro reaction of GriF and ProC *12*
   3.14 In vitro analysis of GriG *12*
   3.15 In vitro analysis of GriH *12*
4. X-Ray crystallography of GriE 13
   4.1 Cloning, expression & purification of GriE for crystallographic experiments *13*
   4.2 Generation of crystallizable constructs *13*
   4.3 Crystallization *13*
   4.4 Data collection, processing, refinement & model building *14*

III. Supplementary Figures **15**

Figure S1. Relative substrate specificities of adenylation domains from the griselimycin megasynthetase. 15
Figure S2. Examples of natural products containing 4-methyl-proline. 16
Figure S3. Biosynthetic routes to 4-MePro. 17
Figure S4. Sequence alignments of related proteins to GriE-H. 19
Figure S5. Feeding experiments to elucidate the biosynthetic origin of the methyl-proline units from griselimycin. 20
Figure S6. LC-MS Analysis of the 4-MePro produced by *Streptomyces lividans* harboring plasmids containing 4-MePro-biosynthesis genes. 21
Figure S7. Overall structure of GriE. 22

Figure S8. Electron density maps for the ligands bound in the GriE structures. 23
Figure S9. Active site geometry of the GriE-ligand complexes. 23
Figure S10. The 6 most probable rotamers of the GriE substrate L-leucine 24
Figure S11. Conformational and B-factor analysis of the GriE crystal structures. 25
Figure S12. Structural comparison of GriE and a homology model of LdoA. 26
Figure S13. Decoding the griselimycin biosynthetic gene cluster sequence. 27
Figure S14. Dotplot of the griA-griH griselimycin biosynthetic gene cluster region. 28
Figure S15. Comparison specificity-conferring residues of (methyl)proline specific adenylation (A) domains of the griselimycin (A2, A5 and A8), nostopeptolide (NosA-A3 and NosD-A2) and nostocyclopeptide (NcpB-A3) megasynthetase 29
Figure S16. Construction of cosmids for heterologous expression of the 4-MePro biosynthesis geneset. 30
Figure S17. Alignment of the five closest homologues in the PDB, which were used for model generation for molecular replacement to the GriE 1-265 sequence. 31
IV. Supplementary Tables 32
Table S1. Genes and encoded proteins of the griselimycin biosynthetic gene cluster. 32
Table S2. Substrate specificity analysis of the 10 adenylation (A) domains from the griselimycin megasynthetase. 33
Table S3. GM and MGM production in cultures of *Streptomyces* DSM 40835 fed with (2*S*,4*R*)-4-methyl-proline. 34
Table S4. X-ray data collection & refinement statistics 34
Table S5. Probes used to screen the *Streptomyces* DSM 40835 cosmid library. 35
Table S6. Plasmids constructed to decipher the griselimycin biosynthetic gene cluster sequence. 35
Table S7. Construction of plasmids for inactivation of the griselimycin biosynthesis pathway. 36
Table S8. Plasmids for the heterlorogous expression of the MePro biosynthetic genes. 37
Table S9. Heterologous expression of MPS genes *in S. lividans* TK24. 37
Table S10. Kinetic parameters determined for GriF. 38
Table S11. Kinetic parameters determined for GriG. 38
Table S12. Primers used for cosmid modification. 38
V. Supplementary References 39

I. Materials

(2*S*,4*R*)-methyl-proline and (2*S*,4*S*)-methyl-proline were a gift from Sanofi-Aventis. pET28b was purchased from Novagen. pColdI was purchased from TAKARA. L-Leucine was purchased from Sigma. All enzymes required for cloning were purchased from Thermo Scientific.

II. Methods

1. Identification and characterization of the griselimycin biosynthetic gene cluster

1.1 Identification and decipherment of the entire griselimycin biosynthetic gene cluster sequence

Our efforts to decipher the griselimycin biosynthetic gene cluster sequence started with a next generation sequencing (NGS) approach. Genomic DNA of the producer strain *Streptomyces* DSM 40835 was extracted according to standard procedures.[1] Whole genome shotgun sequencing was performed at SEQ-IT (Kaiserslautern, Germany) using the 454 technology to obtain 31 sequence scaffolds harboring together 1112 sequence contigs. The sequence data were screened for nonribosomal peptide synthetase (NRPS) encoding pathways using a genome-scanning pipeline provided by Jacques Ravel

(available at: http://nrps.igs.umaryland.edu/nrps/2metdb/Genome_scanning_download.html). Retrobiosynthetic analysis suggest that griselimycins derive from the linear peptide intermediate *N*-Acetyl/*N*-Me-**Val** – **MePro** – *N*-Me-**Thr** – **Leu** – **MePro** – **Leu** – *N*-Me-**Val** – (**Me)Pro** – *N*-Me-**Leu** – **Gly** which (according to textbook biosynthetic logic) is synthesized by a decamodular NRPS assembly line and released via intramolecular cyclization to reveal the griselimycin depsipeptide scaffold. A NRPS-type biosynthetic gene cluster encoding a termination module with predicted substrate specificity for glycine was identified as most likely candidate pathway for griselimycin biosynthesis. As illustrated in Figure S13A this gene cluster (located on Scaffold000017) contained several sequence gaps and upstream sequence information was missing. To elucidate the entire pathway sequence, a cosmid library of the griselimycin producer was constructed and screened for clones harboring parts of the corresponding gene cluster, which allowed subcloning and sequencing of unknown gene cluster fragments and the connection to the adjacent sequence scaffold as shown in Figure S13B/C. However, this approach was complicated due to large sequence repeats within the NRPS encoding region (Figure S14), which required subcloning of cosmid inserts into smaller fragments via multistep procedures as described below. First, a cosmid library of *Streptomyces* DSM 40835 was generated by using pOJ436[2] as cosmid vector according to previously established protocols (e.g. as described by Xu et al.[3]). Robotically produced high-density colony arrays (Hybond N+; Amersham Pharmacia) were utilized for the screening of 2304 cosmid clones with different probes (Probes 1-5) specific for the putative griselimycin gene cluster region. The DNA fragments for the probes were generated by PCR, gel-purified and subsequently labeled using the DIG High Prime DNA Labeling and Detection Kit (Roche). PCRs were performed with Taq polymerase (Fermentas) or Phu polymerase (Finnzymes) using chromosomal DNA from *Streptomyces* DSM 40835 as template and the oligonucleotides indicated in Table S5. Hybridization and detection experiments were performed under standard conditions. Several cosmids hybridizing with different sets of probes were identified and verified by end-sequencing using the oligonucleotides T4 (5'-CACCTGTGGCGCCGGTGATG-3') / T7 (5'-TAATACGACTCACTATAGGG-3'). Based on results from additional restriction analysis cosmids A:N14 and A:I17 were selected for subcloning approaches. By applying standard restriction and ligation methods plasmids pGri2a, pGri3a, pGri4a, pGri5a, pGri7a, pGri9a and pGri10a were generated (Table S6). Plasmids pGri4a, pGri7a, pGri9a and pGri10a were completely sequenced *de novo*. With the obtained data the sequence of the putative griselimycin biosynthetic gene cluster region of Scaffold000017 was completely deciphered. In parallel to the subcloning approaches targeting Scaffold000017 the cosmid library was screened for chromosomal fragments connecting to the 5'end of the cosmid A:N14 insert. Based on the data from cosmid end-sequencing Probe 6 was generated (Table S5) and used for hybridization experiments. Cosmid B:N22 hybridizing with Probe 6, but not with Probes 1-5 was selected for subcloning approaches. Data obtained from cosmid end-sequencing allowed for connection of Scaffold000017 with Scaffold000019. Using conventional cloning methods plasmids pCK-31, pCK-29, pCK-38, pCK-39, pCK-40, pCK-42, pCK-44A and pCK-44F were generated (Table S6) and the subcloned fragments (except from pCK-29 and pCK-31) were completely sequenced to decipher the sequence of the entire griselimycin gene cluster (Figure S13).

1.2 In silico analysis of the griselimycin biosynthetic gene cluster sequence

Sequence analysis of the identified griselimycin biosynthetic pathway was performed with the Geneious software versions 5.3-9.03 (Biomatters, Auckland, New Zealand). Putative gene functions were assigned based on PFAM[4] and BLAST[5] homology searches. Additional *in silico* analysis of the NRPS subunits was carried out using the antiSMASH 3.0[6] and the PKS/NRPS analysis software tools.[7] By *in silico* analysis it was proposed that the griselimycin biosynthesis gene cluster comprises 26 genes (Figure 1A and Table S1; see also Kling *et al.*[8]). The annotated sequence of the griselimycin biosynthetic gene cluster is accessible at GenBank under accession number KP211414.

As illustrated in Figure S14, the NRPS encoding genes *griA* and *griB* contain large repetitive regions explaining the encountered difficulties during the NGS data assembly and the subsequent subcloning approaches to completely decipher the entire griselimycin biosynthetic gene cluster sequence.

1.3 Inactivation of the griselimycin biosynthesis pathway

Insertional mutants of *Streptomyces* DSM 40835 were created according to an approach described by Gust et al. for PCR-targeted *Streptomyces* gene replacement.[9] Gene cluster fragments of about 4.0-5.5 kb in size which are homologues to parts of *griA*, *griC* and the methyl-proline biosynthesis gene set *(griE-H*; designated as *mps* operon*)*, respectively, were amplified from chromosomal DNA of the producer strain using Pfx polymerase (NEB) and the oligonucleotides listed in Table S7. The obtained products were subcloned into pBluescript SK+ to yield plasmids pNRPS-1, pNRPS-3 and pMPS. Subsequently, an *aac(3)IV-oriT* gene disruption cassette (*aac(3)IV* = Apra^R) was amplified from pCK-1 (C. König, unpublished) using Pfx polymerase (NEB) and the oligonucleotides listed in Table S7. The obtained PCR products were used to replace griselimycin gene cluster fragments inserted in plasmids pMPS, pNRPS-1 and pNRPS-3 by applying the Red/ET cloning technology (Genebridges).[10, 11] Experiments were carried out in the Red/ET-proficient strain *E. coli* HS996/pRedET(tet)(Genebridges). The obtained plasmids (pNRPS-1-apra-oriT, pNRPS-3-apra-oriT and pMPS-apra-oriT; Table S7) were then transferred into *Streptomyces* DSM 40835 via conjugation according to standard procedures.[1] Obtained transconjugants growing in the presence of 50 µg/ml apramycin were analyzed for correct genetic integration into the target gene loci via PCR analysis (data not shown). To analyze the generated mutants for griselimycin production the strains were grown in parallel to the wildtype producer. Cultivations were performed at 28 °C in 250 ml shake flasks with 30 ml NL5645 medium (2.0 % lactose, 2.5 % soy meal, 0.5 % NaCl, 0.1 % $CaCO_3$ and 0.5 % yeast extract, pH 6.8; amended with 50 µg/ml apramycin in case of the mutant strains). At different time points the culture supernatant was collected, filtered and mixed with an equal volume of ethanol. After precipitation and separation of undissolved substances the supernatants were analyzed by UHPLC (Ultra High Performance Liquid Chromatography; data not shown). The analysis revealed that griselimycin production was abolished in all three mutant strains indicating that the targeted genetic regions are indeed required for griselimycin biosynthesis.

2. Substrate specificity of adenylation domains

2.1 In silico analysis of adenylation domain substrate specificities

To determine the putative substrate specificity of the adenylation (A) domains *in silico* the ten extracted A domain sequences (designated as A1-A10) from the griselimycin megasynthetase GriABC were analyzed using antiSMASH 3.0[6] (Table S2).

Adenylation domains of the (4*R*)methyl-proline incorporating modules A2, A5 and A8 (the latter preferentially incorporates proline) were analyzed in more detail and compared to (4*S*)methyl-proline and proline specific adenylation domains of two characterized cyanobacterial NRPS pathways: the nostopeptolide (Nos), and the nostocyclopeptide (Ncp) megasynthetases (NosA-A3, NosD-A2 and NcpB-A3).[12, 13] As illustrated in Figure S15, the 8Å signature (a set of 34 active site residues as defined by Rausch *et al.*[14]) as well as the 10 specificity-conferring residues of the A domain binding pocket (defined by Stachelhaus *et al.*[15] and Challis *et al.*[16]) turned out to be very similar. It can be speculated that the more bulky valine residue in position 330 of A8 (versus an alanine residue in the corresponding position of A2 and A5) and the more bulky isoleucine residue in position 331 of NosD-A2 (versus an alanine residue in the corresponding position of NosA-A3 and NcpB-A3) might have an impact on the specificity towards proline.

However, next to the ten active site residues described by Challis *et al.*[16] and Stachelhaus *et al.*[15], substrate specificity might be affected by additional residues located in the binding pocket or in other relevant positions of the A domain. In addition, other catalytic domains involved in subsequent biosynthesis steps (e.g. condensation domains) can contribute to substrate selectivity.

2.2 Biochemical analysis of adenylation domains A2, A5 and A8

To obtain experimental evidence for adenylation (A) domain substrate specificities of (methyl)proline incorporating NRPS modules the A domains of modules 2, 5 and 8 (designated as A2, A5 and A8) were expressed as N-terminal His6-tagged proteins. DNA fragments coding for His-A2 (66.2 kDa), His-A5 (66.1 kDa) and His-A8 (66.9 kDa) were amplified from cosmids A:N14 (A2 and A5) and B:H19 (A8) using Phusion polymerase (ThermoFisher Scientific) and the oligonucleotides A2_for 5'-CCGACCATATGGATCCGGATGTGACGGTGGG-3' and A2_rev 5'-ACCGGGAATTCGCCGACGGCGGGCAGGCCGA-3' (due to the high sequence homology, all three A domains can be amplified by using the same oligonucleotides). The PCR products were subcloned into the pJET1.2/blunt vector (Fermentas) and sequenced to yield plasmids pJETA2, pJETA5 and pJETA8. Subcloning of the verified ~1.8 kb A domain fragments from the pJET1.2/blunt derivatives into the expression vector pET28b(+) via *Nde*I/*Eco*RI revealed plasmids pETA2, pETA5 and pETA8.

All constructs were transformed into *E. coli Rosetta* BL21 (DE3) pLysS/RARE for protein expression. Cultivations were carried out in LB medium supplemented with 50 µg/ml kanamycin and 34 µg/ml chloramphenicol at 37 °C until an OD_{600} of approximately 0.8 was reached. Expression was induced with 0.2 mM isopropyl-β-D-thiogalactopyranoside (IPTG) and cultivation was continued at 16 °C overnight. Cells were harvested by centrifugation (6000 rpm, 10 min, 4 °C), re-suspended in buffer A (20 mM Tris–HCl, pH 7.8, 200 mM NaCl, and 10% [v/v] glycerol) and lysed by French press (2 passes at 700 psi). After removal of the cell debris by centrifugation (21,000 rpm, 10 min, 4 °C) the target proteins could be obtained in the soluble fraction. Prepacked HisTrap™ HP columns (GE Healthcare Life Science) were used for preparative purification of the

histidine-tagged A domains by immobilized metal ion affinity chromatography (IMAC) on the Äkta prime™ plus system (Ge Healthcare). 15 ml protein lysates were filtered through a sterile filter and loaded onto the 1 ml HisTrap column. Purification was performed as recommended in the GE Healthcare manual (HisTrap HP, Instructions 71-5027-68 AF). The target protein was eluted from the column in a stepwise imidazole gradient with buffer B (20 mM Tris–HCl, pH 7.8, 200 mM NaCl, and 10% [v/v] glycerol and 60, 100, 200, 300 and 500 mM imidazole, 10 ml fractions). Fractions containing the pure target protein, as determined by SDS–polyacrylamide gel electrophoresis (PAGE), were combined and concentrated to approximately 200 µl by using Amicon Ultra PL-10 centricons. Then 800 µl of storage buffer (50 mM Tris–HCl, pH 7.5, 50 mM NaCl, and 10% [v/v] glycerol) was added to the concentrated protein before flash-freezing in liquid nitrogen and storage at -80 °C. Protein concentrations were determined using the Bradford assay (Bio-Rad, Hercules, CA, USA).
To determine substrate specificity 100 µl ATP-[^{32}P]PPi reactions[17] containing Tris–HCl (pH 7.5, 75 mM), $MgCl_2$ (10 mM), dATP (5 mM), amino acid (5 mM) and protein (2 µg) were performed at 30 °C. ^{32}P tetrasodium pyrophosphate was obtained from Perkin Elmer (NEN #NEX019). The reactions were started by the addition of [^{32}P]PPi (0.1 µCi final amount) for up to 30 min before quenching with 500 µl charcoal suspension (0.1 M $Na_4P_2O_7$ and 0.35 M perchloric acid in H_2O amended with 1.6 % [w/v] activated charcoal). The charcoal was pelleted by centrifugation prior to being washed twice with 500 µl wash solution (0.1 M $Na_4P_2O_7$ and 0.35 M perchloric acid in H_2O), re-suspended in 500 µl H_2O and counted by liquid scintillation (Beckman LS6500). The experiments were carried out in triplicates for each substrate concentration with a negative control (no amino acid). The results are illustrated in Figure S1.

2.3 Feeding of **(2*S*,4*R*)***-4-methyl-proline to* **Streptomyces** *DSM 40835*

To evaluate if methyl-griselimycin (MGM) production can be increased by feeding 4-methyl-proline, *Streptomyces* DSM40835 was grown in 30 ml NL5429 medium in 100 ml flask at 28 °C for several days. For inoculation of production cultures, 1 ml of the well-grown pre-culture was transferred to 30 ml fresh NL5645 medium supplemented with 0 µg/ml, 20 µg/ml or 200 µg/ml (2*S*,4*R*)-4-methyl-proline. After 1, 2, 3 and 4 days of cultivation, the culture supernatant was collected, filtered and mixed with an equal volume of ethanol. After precipitation and separation of undissolved substances the supernatants were analyzed by UHPLC (Ultra High Performance Liquid Chromatography) for GM and MGM production. The measurements were performed in triplicates and the results are illustrated in Figure 2A and Table S3.

3. Methyl-proline biosynthesis

3.1 Biosynthetic origin of methyl-proline

To elucidate the biosynthetic origin of the griselimycin (2*S*,4*R*)-4-methyl-proline units 50 ml shake flask cultures of *Streptomyces* DSM 40835 were grown at 30 °C in 50 ml production medium (Medium 5288: 1.5% glycerol, 1.0% soy meal, 0.5% NaCl, 0.1% $CaCO_3$ and 0.0001% $CoCl_2$ x 7 H_2O; adjust pH to 6.6 prior sterilization). Solutions of D_3-Methionine (50 mg in 4 ml H_2O) and D_9-Leucine (50 mg in 4 ml H_2O) were fed to the cultures in sixteen portions (250 µl each) during cultivation over 5 days. In parallel, a control experiment was carried out by feeding a culture with the same volumes of pure H_2O without labeled amino acids. Cultures were harvested by centrifugation and the supernatants were subsequently extracted with 50 ml ethyl acetate. After evaporation to

dryness the extracts were re-dissolved in 200 µl methanol and small amounts of griselimycin (~ 100 µg) from each experiment was purified via HPLC. An Agilent 1100 series solvent delivery system that was coupled to Bruker HCTplus ion trap mass spectrometer was used. Chromatographic separation was carried out on an RP column Nucleodur C18 (125 by 2 mm, 3 µm particle size; Macherey and Nagel) that was equipped with a matching precolumn. The mobile phase gradient (solvent A: H_2O+0.1% formic acid and solvent B: acetonitrile + 0.1% formic acid) was linear from 5% B at 2 min to 95% B at 32 min, followed by 3 min with 95% B at a flow rate of 0.4 mL/min. Diode-array detection was carried out at 200-600 nm and mass detection was performed in the positive and negative ionization mode within a range of 100-1200 amu.

Around 100 µg of purified griselimycin from each feeding experiment and the control experiment was amended with 150 µl 6N HCl and incubated at 110 °C für 20 h. The reaction mixtures were dried and re-dissolved in 100 µl coupling solution (acetonitrile:pyridine:triethylamine:water 10:5:2:3). For amino acid derivatization 5 µl of phenylisocyanate reagent was added. After incubation for 5 min at room temperature the reaction mixture was evaporated to dryness. The generated phenylcarbamoyl amino acid derivatives were dissolved in water:acetonitrile (7:2) for HPLC/MS analysis, which was performed using the same HPLC-MS device as mentioned above. Chromatographic separation was carried out on a Luna HydroRP column (100 by 2 mm, 2.5 µm particle size; Phenomenex) that was equipped with a matching precolumn. The mobile phase gradient (solvent A: H_2O+0.1% formic acid and solvent B: H_2O/methanol + 0.1% formic acid) was linear from 15% B at 2 min to 100% B at 17 min, followed by 3 min with 100% B at a flow rate of 0.35 mL/min. Diode-array detection was carried out at 200-600 nm and mass detection was performed in the positive ionization mode within a range of 200-1000 amu. The isotope pattern of the detected amino acid derivatives from the two feeding experiments compared to the unlabeled control was analyzed (Figure S5). The results are illustrated for phenylcarbamoyl-leucine, phenylcarbamoyl-*N*-Me-leucine, phenylcarbamoyl-proline and phenylcarbamoyl-4-Me-proline. The first two derivatives were analyzed as positive control for successful incorporation of D_{10}-leucine (expected for both derivatives) and D_3-methionine (expected for the *N*-Me-leucine derivative). Due to efficient D/H-exchange of the deuterium in α-position of D_{10}-leucine the most significant isotope peak was $[M+Na]^+$ plus 9 amu. As expected, none of the labeled precursors were incorporated into proline, whereas in case of 4-Me-proline a mass shift of 7 amu was observed in the feeding experiment with D_{10}-Leucine. Considering that the original deuterium label in α-position is not detectable due to efficient D/H-exchange the detected isotope pattern correlates with a biogenesis of the griselimycin (2*S*,4*R*)-4-Me-proline units via a similar scenario as proposed for (2*S*,4*S*)-4-Me-proline biosynthesis in the cyanobacterial nostopeptolide pathway[18].

3.2 Complementation of the mps mutant to restore (methyl)griselimycin production

To demonstrate that *griE-H* (also designated as *mps* genes) are relevant for (2*S*,4*R*)-4-methyl-proline biosynthesis the *mps* mutant (generated with plasmid pMPS-apra-oriT as described in 1.3) was complemented (1) by feeding of (2*S*,4*R*)-4-methyl-proline and (2) by genetic complementation via chromosomal integration of a *griE-H* expression construct. Cultivations and (methyl)griselimycin production analysis was carried out as described in 2.3.

In the feeding experiment, cultures of the *mps* mutant were amended with 20 µg/ml (2*S*,4*R*)-4-methyl-proline, which allowed to restore (methyl)griselimycin production

(Figure 2B). In parallel cultivations of the *mps* mutant without supplementation of (2*S*,4*R*)-4-methyl-proline no (methyl)griselimycin could be detected (cp. 1.3).
For the genetic complementation of the *mps* mutant an expression plasmid comprising *griE-H* under the control of the constitutive *PermE* promoter (P(ermE)-mps; C. König, unpublished) was integrated into the ϕC31 phage attachment site of the *mps* mutant strain. As illustrated in Figure 2C griselimycin production could be successfully restored, however, no methylgriselimycin was detected and griselimycin production levels were lower compared to the wildtype producer. This is probably due to lower (2*S,*4*R*)-4-methyl-proline production levels achieved with the P(ermE)-mps expression construct compared to the native pathway.
In summary, both complementation approaches clearly indicate that the *mps* operon is involved in the synthesis of (2*S,*4*R*)-4-methyl-proline, which represents an essential precursor for (methyl)griselimycin biosynthesis.

3.3 Heterologous expression of methyl-proline biosynthesis genes in **S. lividans** *TK24.*

A heterologous expression plasmid was constructed based on the previously obtained cosmid, pOJ436-BL23. pOJ436-BL23-5-ermE, pOJ436-griE_D, F_D, G_D, H_D were constructed using the method described in Figure S16. pOJ436-BL23. pOJ436-BL23-5-ermE, pOJ436-griE_D, F_D, G_D, H_D were digested with KpnI and SphI. The DNA fragment harboring the *gri* genes was cloned into the KpnI and SphI sites of pUWL218 resulting in pUWL-BL23-5-ermE, pUWL-griE_D, F_D, G_D and H_D, respectively.
20 ml of YEME medium containing 5 mg/l of thiostrepton were inoculated with *Streptomyces lividans* TK24 harboring pUWL-MPSs, pUWL-griE_D, pUWL-griF_D, pUWL-griG_D, or pUWL-griH_D and incubated in spring-flasks at 30°C for one week. The cells were harvested by centrifugation and metabolites were extracted with methanol. The methanol was removed by evaporation and the residual material was dissolved in 1 ml of methanol for LC-MS analysis and stereochemistry determination of the produced methyl-proline using Marfey's method[19]. Obtained cell extract was measured by LC-MS analysis equipped with a HILIC column (Phenomenex) and 10 mM ammonium acetate/acetonitrile as mobile phase.
The stereochemistry of methyl-proline was analyzed by using an advanced Marfey's method. 100 µl of cell extract were used for the assay. The solvent was removed by evaporation. Residual material was dissolved in 50 µl of water. The obtained solution was mixed with 20 µl of $NaHCO_3$ and 100 µl of 1 mg/ml 1-fluoro-2,4-dinitrophenyl-5-L-leucinamide (L-FDLA) or D-FDLA. The derivatization reaction was carried out at 37°C for 1 hour before quenching by the addition of 20 µl of 5% HCl. 810 µl of acetonitrile were added to the reaction mixture and obtained solution was analyzed by LC-MS analysis.

3.4 Cloning, expression and purification of GriE

The gene *griE* was amplified by PCR using the forward primer 5'-**CAT*ATG***CAGCTCACGGCCGATCAGGT-3' (boldface letters indicate the NdeI site, italic letters indicate the start codon of GriE) and the reverse primer 5'-**GAATTC**TCATGCCAGCCTCGATTCGG-3' (boldface letters indicate the EcoRI restriction site) and the genome of *Streptomyces* DSM 40835 as a template. The obtained fragments were sub-cloned into the pJET1.2 vector. From the obtained construct, the *griE* gene was excised with NdeI and EcoRI and inserted between the NdeI and EcoRI sites of pET28b, resulting in the expression plasmid pET28-*griE*.

200 ml of LB medium supplemented with kanamycin were inoculated with BL21 (DE3) harboring pET28-*griE*. The culture was incubated at 37˚C until expression of GriE was induced by the addition of 0.1 mM Isopropyl-β-D-thiogalactopyranosid (IPTG) at an OD_{600} of 0.5. The culture was then further incubated at 16˚C over night. Cells were harvested by centrifugation and resuspended in buffer containing 20 mM Tris-HCl (pH 8.0), 200 mM NaCl and 10% glycerol. Lysis was achieved by sonication and cell debris was removed by centrifugation. Recombinant GriE fused with a N-terminal hexa-histidine-tag was purified from the filtrated lysate using Ni-NTA superflow resin (Quiagen). The elution buffer was exchanged with buffer containing 20 mM Tris-HCl (pH 8.0), 200 mM NaCl, and 50% glycerol. The samples were stored at -20˚C.

3.5 Cloning, expression and purification of GriF

The gene *griF* was amplified by PCR using the forward primer 5'-AA**GAATTC***ATG*AGCGATAGGTCTCCGTC-3' (boldface letters indicate EcoRI site, italic letters indicate start codon of GriF) and the reverse primer 5'-TT**AAGCTT**TCAGGTATCGACCGTGGGAAG-3' (boldface letters indicate HindIII) and the genome of *Streptomyces* DSM 40835 as a template. The obtained fragments were cloned into the vector pJET1.2 vector resulting in pJET-*griF*. From the obtained construct, the *griF* gene was excised with EcoRI and HindIII and inserted between the EcoRI and HindIII sites of pColdI, resulting in the expression plasmid pColdI-*griF*.
200 ml of TB medium supplemented with ampicillin were inoculated with BL21 (DE3) harboring pColdI-*griF*. The culture was incubated at 37˚C until an OD_{600} of 0.5 was reached. The culture was then transferred to 15°C and incubated for 30 min. After induction of the GriF expression by the addition of 0.05 mM IPTG, the culture was further incubated for 24 hours. Cells were harvested by centrifugation and resuspended in buffer containing 20 mM Tris-HCl (pH 8.0), 200 mM NaCl, 10% glycerol, 0.1% NP-40 and 10 mM β-mercaptoethanol. Lysis was achieved by sonication and cell debris was removed by centrifugation. Recombinant GriF fused with a N terminal hexa-histidine-tag was purified from the filtrated lysate using Ni-NTA superflow resin (Qiagen). The elution buffer was exchanged with buffer containing 20 mM Tris-HCl (pH 8.0), 200 mM NaCl, 10% glycerol, and 1 mM Dithiothreitol (DTT). The samples were stored at -80˚C.

3.6 Cloning, expression and purification of GriG

The gene *griG* was amplified by PCR using the forward primer 5'-AA**CAT*ATG***AGCAGTGATCACTTTGG -3' (boldface letters indicate EcoRI site, italic letters indicate start codon of GriG) and the reverse primer 5'-**TTAAGCTT**TTACAATTTCCCTGGTGCGG -3' (boldface letters indicate HindIII) and the genome of *Streptomyces* DSM 40835 as a template. The obtained fragments were cloned into the vector pJET1.2 vector resulting in pJET-*griG*. From the obtained construct, the *griF* gene was excised with EcoRI and HindIII and inserted between the EcoRI and HindIII sites of pColdI, resulting in the expression plasmid pColdI-*griG*.
200 ml of TB medium supplemented with ampicillin were inoculated with BL21 (DE3) harboring pColdI-*griG*. The culture was incubated at 37˚C until an OD_{600} of 0.5 was reached. The culture was then transferred to 15°C and incubated for 30 min. After induction of the GriG expression by the addition of 0.05 mM IPTG, the culture was further incubated for 24 hours. Cells were harvested by centrifugation and resuspended in buffer containing 20 mM Tris-HCl (pH 8.0), 200 mM NaCl and 10% glycerol. Lysis was achieved by sonication and cell debris was removed by centrifugation. Recombinant GriG fused with a N terminal hexa-histidine-tag was purified from the filtrated lysate

using Ni-NTA superflow resin (Qiagen). The elution buffer was exchanged with buffer containing 20 mM Tris-HCl (pH 8.0), 200 mM NaCl and 50% glycerol. The samples were stored at -20°C.

3.7 Cloning, expression and purification of GriH

The gene *griH* was amplified by PCR using the forward primer 5'-AA**GAATTC***ATG*ACTCGAAGGATGCGCTATG -3' (boldface letters indicate EcoRI site, italic letters indicate start codon of GriH) and the reverse primer 5'-TT**AAGCTT**TTACAATTTCCCTGGTGCGG -3' (boldface letters indicate HindIII) and the genome of *Streptomyces* DSM 40835 as template. The obtained fragments were cloned into the vector pJET1.2 vector resulting in pJET-*griF*. From the obtained construct, the *griF* gene was excised with EcoRI and HindIII and inserted between the EcoRI and HindIII sites of pColdI, resulting in the expression plasmid pColdI-*griH*.
The DNA fragment encoding for GriH fused with a N-terminal histidine tag was amplified by PCR by using pColdI-*griH* as a template and placed under the control of a synthetic promoter constructed by Siegl *et al.*[20] by overlap extension PCR. The obtained DNA fragment was cloned into EcoRI and XbaI site of pUWL218 resulting in pUWL-LAGriH.
200 ml of YEME medium containing thiostrepton were inoculated with *Streptomyces lividans* TK24 harboring pUWL-LAGriH. The culture was incubated in a spring-flask at 30°C for one week. After incubation for 30 min at 4°C, the cells were harvested by centrifugation and resuspended in buffer containing 20 mM Tris-HCl (pH 8.0), 200 mM NaCl, 10% glycerol, 0.1% NP-40 and 10 mM β-mercaptoethanol and several mg of lysozyme. Lysis was achieved by sonication and cell debris was removed by centrifugation. Recombinant GriH fused with a N terminal hexa-histidine-tag was purified from the filtrated lysate using Ni-NTA superflow resin (Qiagen). The elution buffer was exchanged with buffer containing 20 mM Tris-HCl (pH 8.0), 200 mM NaCl, 10% glycerol, and 1 mM Dithiothreitol (DTT). The samples were stored at -80°C.

3.8 Cloning, expression and purification of ProC (NC_010473.1, residues 343390 to 344199) from E. coli

proC was amplified by PCR using the forward primer 5'-AA**CAT*ATG***GAAAAGAAAATCGGTTTTATTGGCTG -3' (boldface letters indicate the NdeI site, italic letters indicate the start codon of GriE) and the reverse primer 5'-TT**GAATTC**AGGATTTGCTGAGTTTTTCTG -3' (boldface letters indicate the EcoRI restriction site) and the genome of *E. coli* DH10β as a template. The obtained fragments were sub-cloned into the pJET1.2 vector. From the obtained construct, the *proC* gene was excised with NdeI and EcoRI and inserted between the NdeI and EcoRI sites of pET28b, resulting in the expression plasmid pET28-*proC*.
200 ml of LB medium supplemented with kanamycin were inoculated with BL21 (DE3) harboring pET28-*proC*. The culture was incubated at 37°C until expression of ProC was induced by the addition of 0.1 mM Isopropyl-β-D-thiogalactopyranosid (IPTG) at an OD_{600} of 0.5. The culture was then further incubated at 16°C over night. Cells were harvested by centrifugation and resuspended in buffer containing 20 mM Tris-HCl (pH 8.0), 200 mM NaCl and 10% glycerol. Lysis was achieved by sonication and cell debris was removed by centrifugation. Recombinant ProC fused with a N-terminal hexa-histidine-tag was purified from the filtrated lysate using Ni-NTA superflow resin (QIAGEN). The elution buffer was exchanged with buffer containing 20 mM Tris-HCl (pH 8.0), 200 mM NaCl, and 10% glycerol. The samples were stored at -80°C.

3.9 *In vitro analysis of GriE*

The reaction mixture containing 0.5 mM leucine (or other amino acids), 1 mM α-ketoglutarate, 25 μM $FeSO_4$, 0.1 mM ascorbate, 0.5 mM DTT, 0.1 mM potassium phosphate buffer (pH8.0) and 2 μM of GriE in a total volume of 50 μl was incubated at 37˚C for 3 hours before quenched by the addition of 50 μl of methanol. Precipitated protein was removed by centrifugation and the supernatant was transferred to a new tube. Produced hydroxyleucine was derivatized using PITC (phenylisothiocyanate) prior to LC-MS analysis by method reported by Palego *et al.*[21]. MeOH and water were removed under a stream of nitrogen and the sample was dissolved in 100 μl of solution A (methanol/1 M sodium acetate/ triethylamine=2/2/1). Solvent was removed under a stream of nitrogen and residual material was dissolved in solution B (methanol/water/triethylamine/PITC=7/1/1/1) and incubated at room temperature for 30 minutes. The solvent was removed by evaporation and residual material was dissolved in 100 μl of methanol for LC-MC analysis. LC-MS analysis was carried out using Agilent equipped with a Luna 2.5u C18(2)-HST 200 x 2.00 mm 2.5 micron (Penomenex). The products were eluted with liner water-acetonitrile (0.1% formic acid) gradient. MS was detected by using HCT (Bruker Daltonics).

3.10 Production of hydroxyleucine in BL21 (DE3) harboring pET28-GriE

200 ml of LB medium containing the kanamycin were inoculated with BL21 (DE3) harboring pET28-GriE and incubated at 37˚C until OD_{600} reached 0.5. 0.1 mM IPTG was added to induce the expression of GriE. The bacteria were further incubated at 16˚C over night. The cells were harvested by centrifugation and resuspended in 200 ml of M9 medium containing 2% glucose and 3 mM leucine. The reaction was carried out for 2 days and cells were removed by centrifugation. The supernatant was washed two times with ethyl acetate. The water layer was applied to 30 g of DOWEX-50W anion exchange resin (Sigma). The resin was first washed with water and hydroxyleucine was eluted with 0.1 M NH_4. NH_4 and water were removed by evaporation. The residual material was dissolved in methanol. Hydroxyleucine was further purified using normal phase chromatography with cellulose and butanol/acetic acid/water=60/15/25. The fractions containing hydroxyleucine were combined and the solvent was removed by evaporation. Residual material was dissolved in water and purified by semi-preparative HPLC equipped with a Luna 5u C18 250 x 10 mm 100 Å (Penomenex) using water/methanol/formic acid=98/2/0.1. The ^{1}H NMR of obtained hydroxyleucine was compared with previously reported ^{1}H NMR of 5-hydroxyleucine[18, 22].

3.11 NMR data of 5-hydroxyleucine

Isolated (2*S*,4*R*)-5-hydroxyleucine, ^{1}H NMR 700 M Hz (700 MHz, D_2O) δ 3.72 (t, *J* = 6.3 Hz , 1H), 3.43 (dd, *J* = -11.2, 5.6 Hz, 1H), 3.39 (dd, *J* = -10.5, 6.3 Hz, 1H), 1.86 (m, 1H), 1,74 (m, 1H), 1.62 (m, 1H), 0.90 (d, *J* = 6.3, 3H).
Reported (2*S*,4*S*)-5-hydroxyleucine, ^{1}H NMR (300 MHz, D_2O) δ 3.77 (dd, *J* = 10, 4 Hz, 1H), 3.47-3.53 (m, 2H), 1.74-1.88 (m, 3H), 0.99 (d, *J* = 6.2 Hz, 3H).[18]
Reported (2*S*,4*R*)-5-hydroxyleucine, ^{1}H NMR (300 MHz, D_2O) δ 3.80 (t, *J* = 6.6 Hz, 1H), 3.53 (dd, *J* = -11.1, 5.7 Hz, 1H), 3.49 (dd, *J* = -11.1, 6.3 Hz, 1H) 1.95 (m, 1H), 1.84 (m, 1H), 1.71 (m, 1H), 1.00 (d, *J* = 6.7 Hz, 3H).[18]

3.12 In vitro analysis of GriF

The reaction mixture containing 0.5 mM (2*S*,4*R*)-5-hydroxyleucine, which was isolated from BL21 (DE3) overexpressing GriE (see 3.10), 1 mM $ZnSO_4$, 2 mM NAD^+, 0.5 mM DTT, 0.1 mM of glycine buffer (pH 10) and 0.6 µM of GriE in total volume of 50 µl was incubated at 30°C for 12 hours before quenched by adding 50 µl 5% trifluoroacetic acid (TFA). Precipitated protein was removed by centrifugation and supernatant was directly applied to LC-MS analysis equipped with a Luna 2.5u C18(2)-HST 200 x 2.00 mm 2.5 micron column (Phenomenex). For the analysis of the kinetic parameters of the GriF reaction, 100 µl reaction mixture containing 100 mM glycine buffer (pH 10), 2 µM GriF, 5 mM NAD^+ and 2-2000 µM hydroxyleucine was incubated at 30°C. The initial velocity of the reaction was estimated by monitoring the increase in absorbance at 340 nm (Table S.10).

3.13 In vitro reaction of GriF and ProC

The reaction mixture containing 0.5 mM (2*S*,4*R*)-5-hydroxyleucine, 1 mM $ZnSO_4$, 2 mM NAD^+, 0.1 mM of glycine buffer (pH 10), 0.6 µM of GriE and ProC in total volume of 50 µl was incubated at 30°C for 12 hours before quenched by adding 50 µl methanol. Precipitated protein was removed by centrifugation and the supernatant was removed by evaporation. Residual material was dissolved in 5 µl of water. 2 µl of 1 M $NaHCO_3$ and 10 µl of 1 mg/ml D-FDLA were added to the solution. The derivatization reaction was carried out at 37°C for 1 hour. The reaction was quenched by adding 20 µl of 5% HCl. The mixture was further mixed with 81 µl acetonitrile and analyzed by LC-MS analysis.

3.14 In vitro analysis of GriG

The kinetic values of esterase activity of GriG were obtained according to the methods reported by Buntin *et al.* using *p*-nitrophenyl acetate as a substrate.[23] The kinetic values were calculated using the Hanes-Woolf plot (Table. S11).

3.15 In vitro analysis of GriH

GriH was analyzed *in vitro* using a reaction coupled to GriF. The reaction was supplemented with cell extract of *Streptomyces* DSM 40835 as it was supposed to contain the potentially required cofactor F420. The mixture containing 0.5 mM (2*S*,4*R*)-5-hydroxyleucine, 1 mM $ZnSO_4$, 2 mM NAD^+, 0.1 mM glycine buffer (pH 10), 10 µl cell extract of *Streptomyces* DSM 40835, 0.6 µM of GriF and 2 µM GriH in a total volume of 50 µl was incubated at 30°C for 12 hours before quenched by adding 50 µl methanol. Precipitated protein was removed by centrifugation and the supernatant was removed by evaporation. Residual material was dissolved in 5 µl of water. 2 µl of 1 M $NaHCO_3$ and 10 µl of 1 mg/ml D-FDLA were added to the solution. The derivatization reaction was carried out at 37°C for 1 hour. The reaction was quenched by adding 20 µl of 5% HCl. The mixture was further mixed with 81 µl acetonitrile and analyzed by LC-MS analysis.
Extraction of F420 from *Streptomyces* DSM 40853 which is deficient in 4-MePro production was achieved by the method reported by Isabelle *et al.*[24].

4. X-Ray crystallography of GriE

4.1 Cloning, expression & purification of GriE for crystallographic experiments

For crystallography purposes, the *griE* gene was cloned into a modified pCOLADuet-1 vector encoding for a N-terminal Strep-tag II and TEV-protease recognition site. The protein was heterologously expressed in *E. coli* BL21 (DE3) in ZYM-5052 auto-inducing medium[25] at 20 °C for 20-24 hr. The cell pellet was resuspended in Tris-HCl (pH 7.5, 20 mM)/NaCl (150 mM) and lysed by sonication. The protein was isolated from the supernatant after centrifugation using a self-packed 10 mL column with Strep-Tactin Superflow High Capacity (IBA) and eluted from the column with a single step of 5 mM d-desthiobiotin. The affinity tag was cleaved off with TEV protease (1:50 mg/mg) at 4 °C overnight. Gel filtration was carried out as final polishing step using a HiPrep Superdex 200 16/60 GL column (GE Healthcare) in Tris-HCl (pH 7.5, 20 mM)/NaCl (150 mM). All chromatographic steps were carried out using an Äkta Purifier system (GE Healthcare). The samples were analyzed by SDS-PAGE (12%), and protein concentrations were determined from the absorbances at 280 nM with the extinction coefficients as calculated by Protparam[26].

4.2 Generation of crystallizable constructs

Crystallizing truncations of GriE were obtained by screening of full-length GriE with proteases from the kits Proti-ACE 1/2 (Hampton Research) according to the manufacturer's manual. Conditions containing the endoproteinase GluC that lead to reproducible crystals were optimized and affinity purified full-length GriE was incubated with GluC (1:100 mg/mg) for 4 hours at room temperature.

The obtained fragments were further purified using a MonoQ 10/10 anion exchanger (GE Healthcare) with a linear gradient from 0 – 1 M NaCl in Tris-HCl (pH 7.5, 20 mM), followed by gel filtration with a HiPrep Superdex 200 16/60 GL column (GE Healthcare) in Tris-HCl (pH 7.5, 20 mM)/NaCl (150 mM).

The fragments present in the main peak fractions from the gel filtration were identified using mass spectrometry and N-terminal peptide sequencing. C-terminal truncations resulting from protease digestion were mimicked by the introduction of stop codons into the plasmid harboring the full-length *griE* gene using site-directed mutagenesis according to the QUIK-Change II protocol (Stratagene/Agilent). The shortened constructs were expressed and purified following the same strategy as applied for the full-length protein.

4.3 Crystallization

Crystallization trials were set up at room temperature with a HoneyBee 961 crystallization robot (Digilab Genomic Solutions) in Intelli 96-3 plates (Art Robbins Instruments) with 200 nL protein solution at different concentrations and 200 nL reservoir solution.

All crystals were obtained for a C-terminally truncated construct comprising the residues 1-265. A crystal of the apo protein (10 mg/ml, incubated at 12°C) was obtained in 20.8 % (w/v) PEG 4000, 0.233 M $LiSO_4$, 0.1 M HEPES/NaOH pH 7.5 and cryo-protected by quick-soaking in the reservoir solution with added 20 % (v/v) glycerol. Ligand complexed crystals were obtained by co-crystallization: 75 µl protein (20 mg/ml in 20 mM Tris/HCl, 150 mM NaCl) were mixed with 10 µl of a solution containing either 10 mM $CoCl_2$ or 10 mM $MnCl_2$, with the latter solution containing 20 mM sodium

ascorbate as stabilizer. After 10 minutes of incubation on ice, 15 µl of a mixture containing 67 mM L-leucine, 67 mM α-ketoglutarate, 67 mM DTT and 730 mM Tris/HCl (pH 9) with a final pH of 8.0 were added. The samples used for co-crystallization thus contained 15 mg/ml protein, 10 mM L-leucine, 10 mM α-ketoglutarate, 10 mM DTT, 112.5 mM NaCl, 125.5 mM Tris/HCl and 1 mM $CoCl_2$ or 1 mM $MnCl_2$ and 2 mM Na-ascorbate.
A crystal containing $CoCl_2$ in the mixture was grown at 20°C in 24.8 % PEG 4000, 16.1 % PEG 200 and 0.1 M HEPES/NaOH pH 7.3 and was cryo-protected by quick-soaking in the reservoir solution with added 20 % (v/v) glycerol.
The protein was co-crystallized with the mixture containing $MnCl_2$ at 20°C in condition F1 of the MORPHEUS crystallization screen[27] (Molecular Dimensions) and did not require any further cryo-protection.

4.4 Data collection, processing, refinement & model building

A dataset of apo GriE was recorded in house at a Micromax 007 HF rotating Cu anode (Rigaku) equipped with a Saturn 944+ CCD detector (Rigaku). Datasets of the ligand-containing crystals were recorded at the beamline X06DA (PXIII) at the Swiss Light Source, Paul Scherrer Institut, Villigen, Switzerland at a wavelength of λ = 1.0 Å. All datasets were recorded at a temperature of 100 K.
The datasets were processed using the Autoproc[28] toolbox (Global Phasing) executing XDS[29], Pointless[30], and Aimless[31]. High resolution cutoffs of 1.82 Å for the apo form, 1.76 Å for the Co^{2+} containing sample and 1.53 Å for the crystals grown in the presence of Mn^{2+} were determined based on CC1/2[32] and I/σI values (Table S4). Molecular replacement was carried out using Phaser[33] from the Phenix suite[34] version 1.10.1 with a search model generated from the five closest homologues in the PDB (Fig. S17) using the Phenix tools Ensembler and Sculptor. The structural models were built using Coot[35] and crystallographic refinement was performed with Phenix.refine[36] including the addition of hydrogens in riding positions and TLS-refinement. Figures of crystal structures were prepared using the PyMOL Molecular Graphics System version 1.8.2.3 (Schrödinger, LLC). Ligand interaction diagrams were generated using the corresponding tool from Maestro version 10.6.014 (Schrödinger, LLC). The homology model of LdoA from *Nostoc punctiforme* was generated using the Phyre2 server[37].

III. Supplementary Figures

A

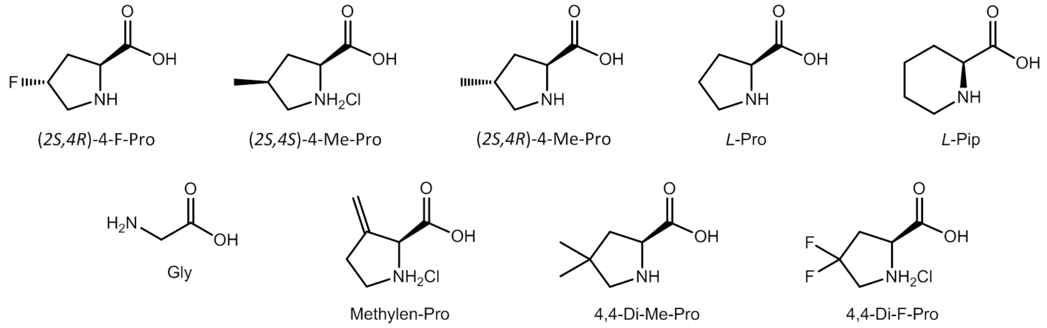

B

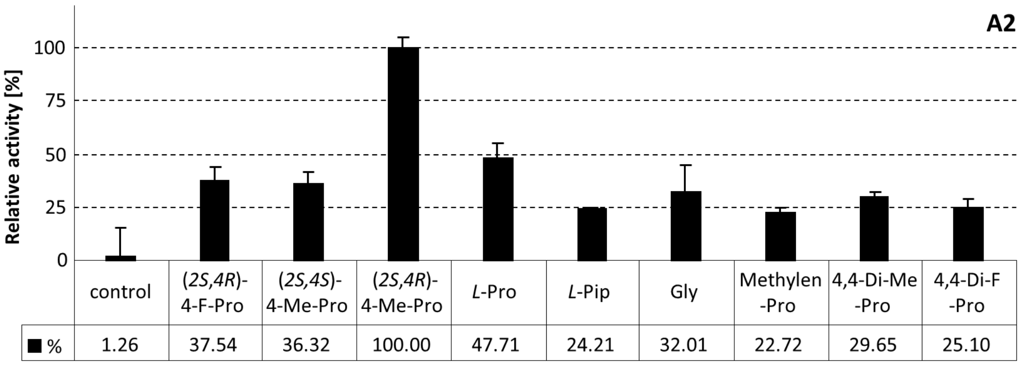

	control	(2S,4R)-4-F-Pro	(2S,4S)-4-Me-Pro	(2S,4R)-4-Me-Pro	L-Pro	L-Pip	Gly	Methylen-Pro	4,4-Di-Me-Pro	4,4-Di-F-Pro
■ %	1.26	37.54	36.32	100.00	47.71	24.21	32.01	22.72	29.65	25.10

Substrate [amino acid]

C

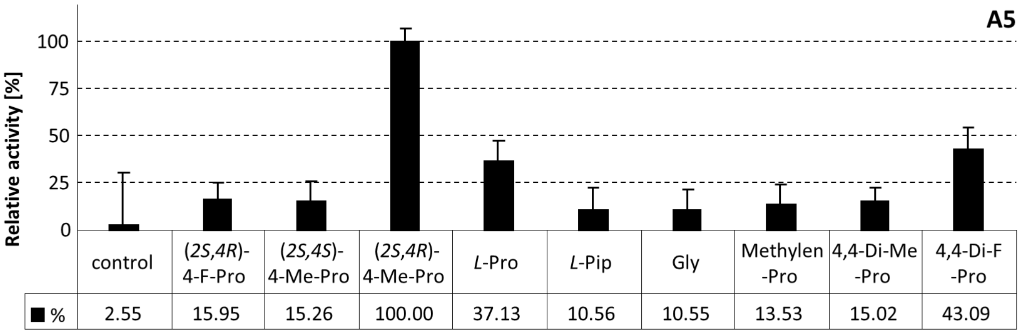

	control	(2S,4R)-4-F-Pro	(2S,4S)-4-Me-Pro	(2S,4R)-4-Me-Pro	L-Pro	L-Pip	Gly	Methylen-Pro	4,4-Di-Me-Pro	4,4-Di-F-Pro
■ %	2.55	15.95	15.26	100.00	37.13	10.56	10.55	13.53	15.02	43.09

Substrate [amino acid]

D

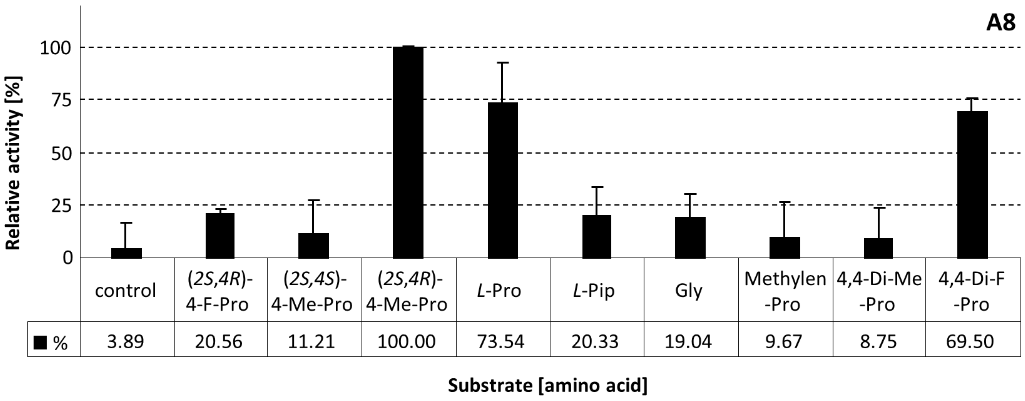

	control	(2S,4R)-4-F-Pro	(2S,4S)-4-Me-Pro	(2S,4R)-4-Me-Pro	L-Pro	L-Pip	Gly	Methylen-Pro	4,4-Di-Me-Pro	4,4-Di-F-Pro
■ %	3.89	20.56	11.21	100.00	73.54	20.33	19.04	9.67	8.75	69.50

Substrate [amino acid]

Figure S1. Relative substrate specificities of adenylation domains from the griselimycin megasynthetase.

(**A**) Chemical structures of substrates used for the ATP-PP$_i$ exchange assays. (**B**)-(**D**) Results obtained with adenylation (A) domains of module 2 (A2), module 5 (A5) and module 8 (A8) in the ATP-PP$_i$ exchange assays with different amino acids and a control without amino acid. The highest activities were set at 100%.

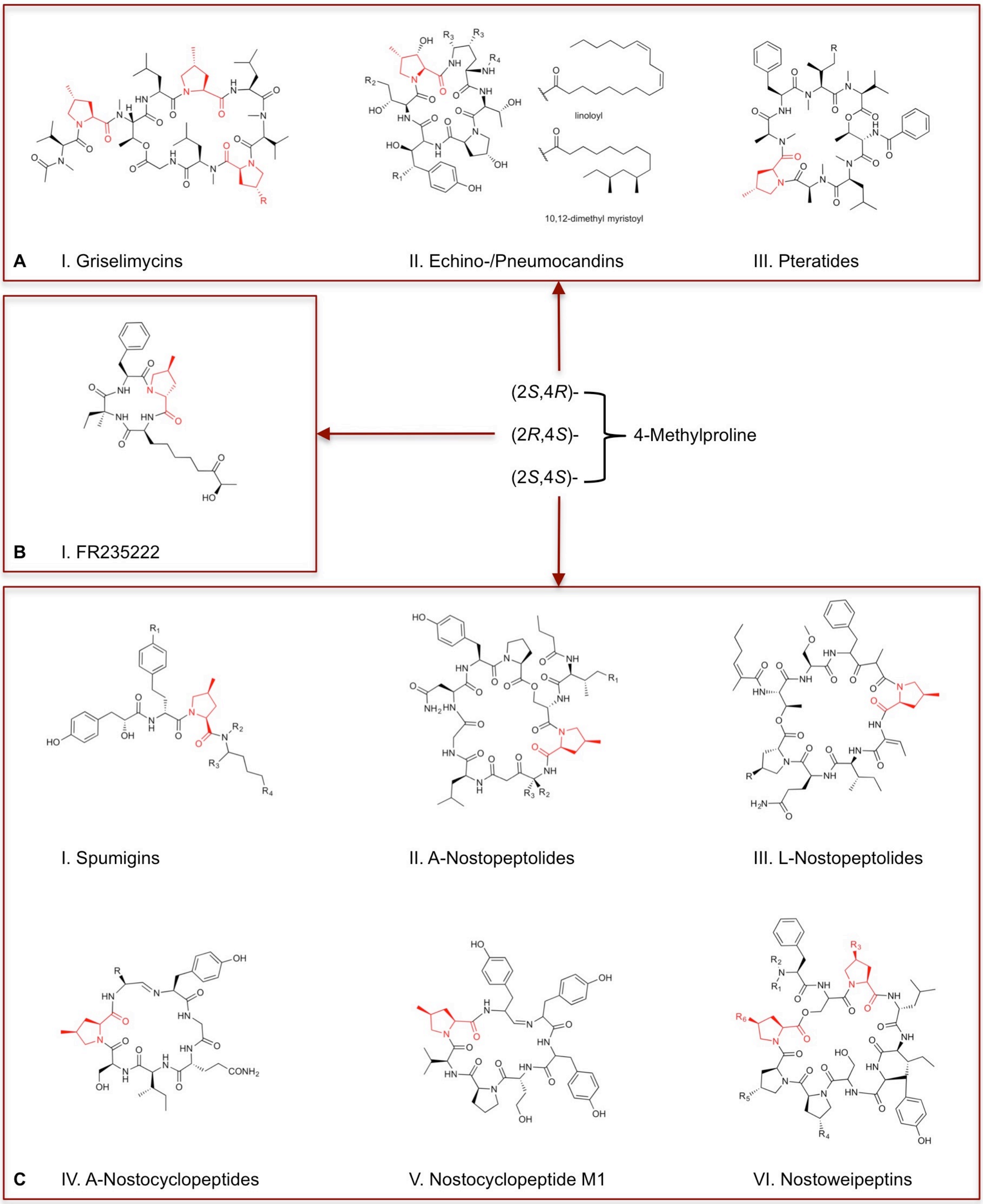

Figure S2. Examples of natural products containing 4-methyl-proline.
(**A**) Compounds incorporating the (2*S*,4*R*)-diastereomer. (I) Griselimycin[8]: R = H; Methylgriselimycin[8]: R = CH_3. (II) Echinocandin B[38-40]: R_1 = OH, R_2 = H, R_3 = OH, R_4 = linoloyl; Echinocandin C[38-40]: R_1 = H, R_2 = H, R_3 = OH, R_4 = linoloyl; Echinocandin D[38-40]: R_1 = H, R_2 = H, R_3 = H, R_4 = linoloyl; Pneumocandin A0[41]: R_1 = OH, R_2 = $CONH_2$, R_3 = OH, R_4 = 10,12-dimethyl myristoyl. (III) Pteratide I[42]: R = H; Pteratide II[42]: R = CH_3. (**B**) FR235222[43] contains (2*R*,4*S*)-4-methyl-proline as a building block. (**C**) Substances containing (2*S*,4*S*)-4-methyl-proline. (I) Spumigin A[44]: R_1 = OH, R_2 = H, R_3 = CH_2OH, R_4 = guanidino; Spumigin B_1/B_2[44]: R_1 = OH, R_2 = H, R_3 = COOH, R_4 = guanidino; Spumigin E[45]: R_1 = OH, R_2 = H, R_3 = CHO, R_4 = guanidino; Spumigin G[45]: R_1 = H, R_2 = H, R_3 = CHO, R_4 = guanidino; Spumigin J[46]: R_1 = OH, R_2 = CH_3, R_3 = COOH, R_4 = NH_2. (II) Nostopeptolide A1[13]: R_1 = CH_3, R_2 = $CH_2CH(CH_3)_2$, R_3 = H; Nostopeptolide A2[13]: R_1 = H, R_2 = $CH_2CH(CH_3)_2$, R_3 = H; Nostopeptolide A3[13]: R_1 = CH_3, R_2 = H, R_3 = $CH_2CH(CH_3)_2$. (III) Nostopeptolide L1[47]: R = OH; Nostopeptolide L2[47]: R = H. (IV) Nostocyclopeptide A1[48]: R = isopropyl; Nostocyclopeptide A2[48]: R = phenyl; Nostocyclopeptide A3[48]: R = p-methylphenyl. (V) Nostocyclopeptide M1[47]. (VI) Nostoweipeptin

W1[47]: R_1 = CH_3CO, R_2 = CH_3, R_3 = CH_3, R_4 = OH, R_5 = OH, R_6 = CH_3; Nostoweipeptin W2[47]: R_1 = CH_3CO, R_2 = CH_3, R_3 = CH_3, R_4 = OH, R_5 = H, R_6 = CH_3; Nostoweipeptin W3[47]: R_1 = CH_3CO, R_2 = CH_3, R_3 = CH_3, R_4 = H, R_5 = OH, R_6 = CH_3; Nostoweipeptin W4[47]: R_1 = CH_3CO, R_2 = H, R_3 = CH_3, R_4 = OH, R_5 = OH, R_6 = H; Nostoweipeptin W5[47]: R_1 = CH_3CO, R_2 = CH_3, R_3 = CH_3, R_4 = OH, R_5 = OH, R_6 = H; Nostoweipeptin W6[47]: R_1 = CH_3CO, R_2 = CH_3, R_3 = H, R_4 = OH, R_5 = OH, R_6 = CH_3; Nostoweipeptin W7[47]: R_1 = H, R_2 = CH_3, R_3 = H, R_4 = OH, R_5 = OH, R_6 = CH_3.

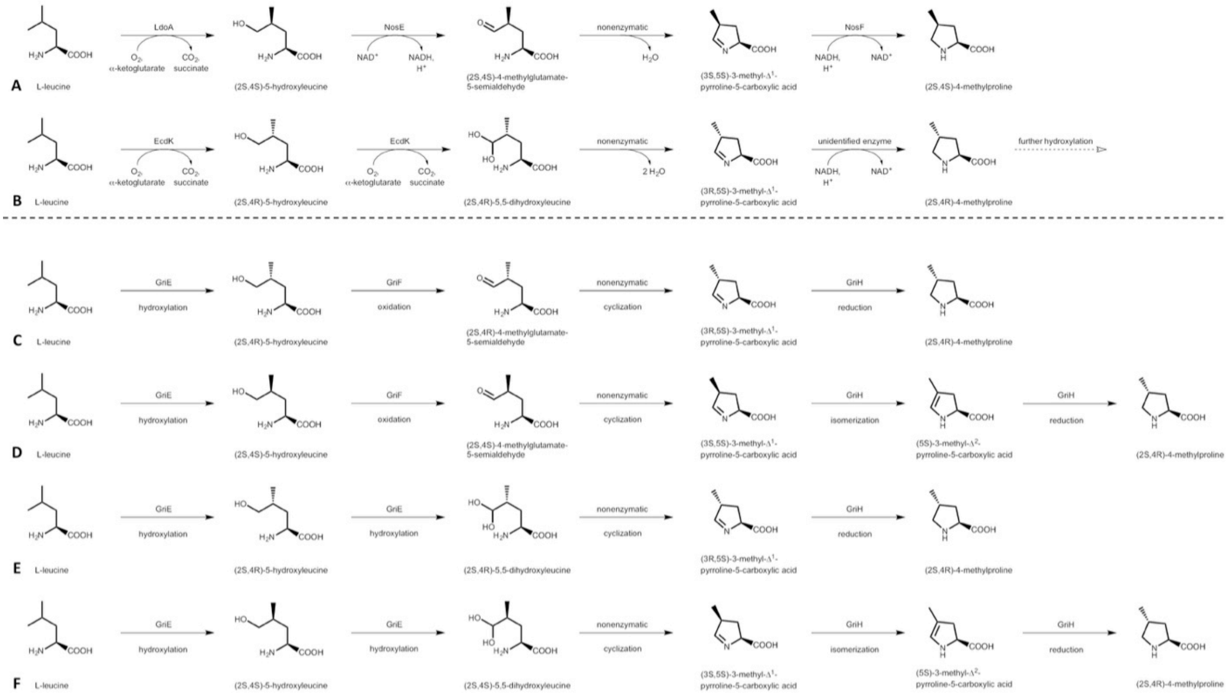

Figure S3. Biosynthetic routes to 4-MePro.

(**A**) Biosynthesis of (2*S*,4*S*)-4-MePro by *Nostoc punctiforme.*[18, 49] (**B**) Formation of (2*S*,4*R*)-4-MePro in the biosynthesis of (2*S*,3*S*,4*S*)-4-methyl-3-hydroxyproline by *Emericella rugulosa*[50]. (**C**) Hypothetical scenario 1 for (2*S*,4*R*)-4-methyl-proline formation in griselimycin biosynthesis. The reaction proceeds analogous to (A) with the hydroxylation of L-leucine as the stereochemistry determining step. (**D**) Hypothetical scenario 2 for (2*S*,4*R*)-4-methyl-proline formation in griselimycin biosynthesis. The first step catalyzed by GriE would generate the same stereoisomer of 5-hydroxyleucine as in (A) and an additional isomerase activity of GriH would be required to obtain the correct product (2*S*,4*R*)-4-methyl-proline. The presence of this additional isomerase activity has to be considered as HrmD from the hormaomycin biosynthesis has been predicted to also catalyze the isomerization of 1-pyrroline derivatives to 2-pyrroline derivatives prior to the reduction of the double bond[51]. (**E**) Hypothetical scenario 3 for (2*S*,4*R*)-4-methyl-proline formation in griselimycin biosynthesis. Analogous to EcdK in echinocandin biosynthesis (B), GriE would catalyze two subsequent hydroxylation steps to (2*S*,4*R*)-5,5-hydroxyleucine. GriF would thus not be involved in this pathway. (**F**) Hypothetical scenario 4 for (2*S*,4*R*)-4-methyl-proline formation in griselimycin biosynthesis. As in scenario 3 (E), GriE would catalyze two subsequent hydroxylation reactions but the stereochemistry of the generated (2*S*,4*S*)-5,5-hydroxyleucine would as in scenario 2 (D) require an additional isomerase activity of GriH to obtain the correct product (2*S*,4*R*)-4-methyl-proline.

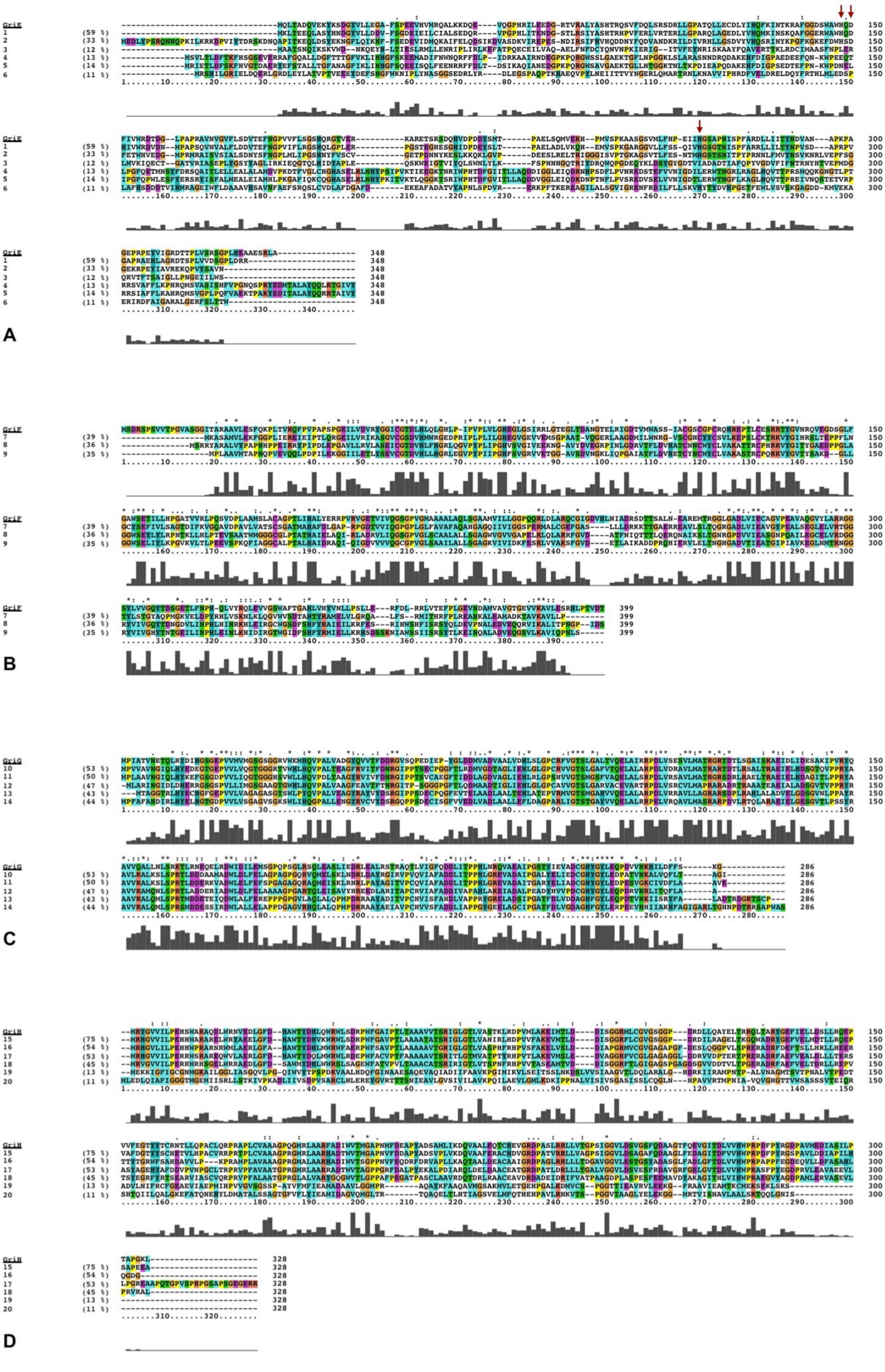
GriE
A
GriF
B
GriG
C
GriH
D

Figure S4. Sequence alignments of related proteins to GriE-H.

The sequence identities of the aligned sequences to the corresponding protein are given in parentheses. (**A**) Sequence alignment to GriE. The residues involved in metal coordination are marked by arrows above the sequence. 1: Phytanoyl-CoA dioxygenase from *Salinispora arenicola* (NCBI: WP_012184556)[52]; 2: Ectoine hydroxylase EctD from *Virgibacillus salexigens* (NCBI: Q2TDY4)[53]; 3: L-Leucine 5-hydroxylase (LdoA) from the nostopeptolide producer *Nostoc punctiforme* (NCBI: ACC80786)[49]; 4: L-Leucine 5-hydroxylase (EcdK) from the echinocandin producer *Emericella rugulosa* (NCBI: AFT91382)[50, 54]; 5: L-Leucine 5-hydroxylase (GloC) from *Glarea lozoyensis* (NCBI: EPE34347)[55, 56]; 6: L-Proline 3-hydroxylase from *Streptomyces sp.* (NCBI: AB007189)[57]. (**B**) Sequence alignment to GriF. 7: 5-exo-Hydroxycamphor dehydrogenase from a *Peptococcaceae* strain (NCBI: KLU60665)[58]; 8: Zn-dependent threonine dehydrogenase from *Chthonomonas calidirosea* (NCBI: WP_016481836)[59]; 9: (2S,4S)-5-hydroxyleucine dehydrogenase NosE from the nostopeptolide producer *Nostoc sp.* GSV224 (NCBI: AAF17283)[13]. (**C**) Sequence alignment to GriG. 10: LipE from laspartomycin producer *Streptomyces viridochromogenes* (NCBI: ELS51230)[60, 61]; 11: LipE from the friulimicin producer *Actinoplanes friuliensis* (NCB: WP_023362363)[62]; 12: LptH from *Streptomyces fradiae* (NCBI: AAZ23080)[63]. 13: CinE from the cinabarramide producer *Streptomyces sp.* JS360 (NCBI: CBW54675)[64]; 14: SalF from the salinosporamide producer *Salinispora tropica* (NCBI: ABP73650)[65]. (**D**) Sequence alignment to GriH. 15: LmbY from the lincomycin producer *Streptomyces lincolnensis* (NCBI: ABX00622)[66]; 16: SibT from the sibiromycin producer *Streptosporangium sibiricum* (NCBI: ACN39743)[67]; 17: TomJ from the tomaymycin producer Streptomyces achromogenes (NCBI: ACN39023)[68]; 18: HrmD from the hormaomycin producer Streptomyces griseoflavus (NCBI: AEH41801)[51]; 19: Pyrroline-5-carboxylate reductase ProC from proline biosynthesis in *E. coli* (NCBI: P0A9L8)[69]; 20: (3S,5S)-3-Methyl-Δ^1-pyrroline-5-carboxylate reductase NosF from from the nostopeptolide producer *Nostoc sp.* GSV224 (NCBI: AAF17284)[13].

The alignments were generated using Clustax X 2.1[70, 71].

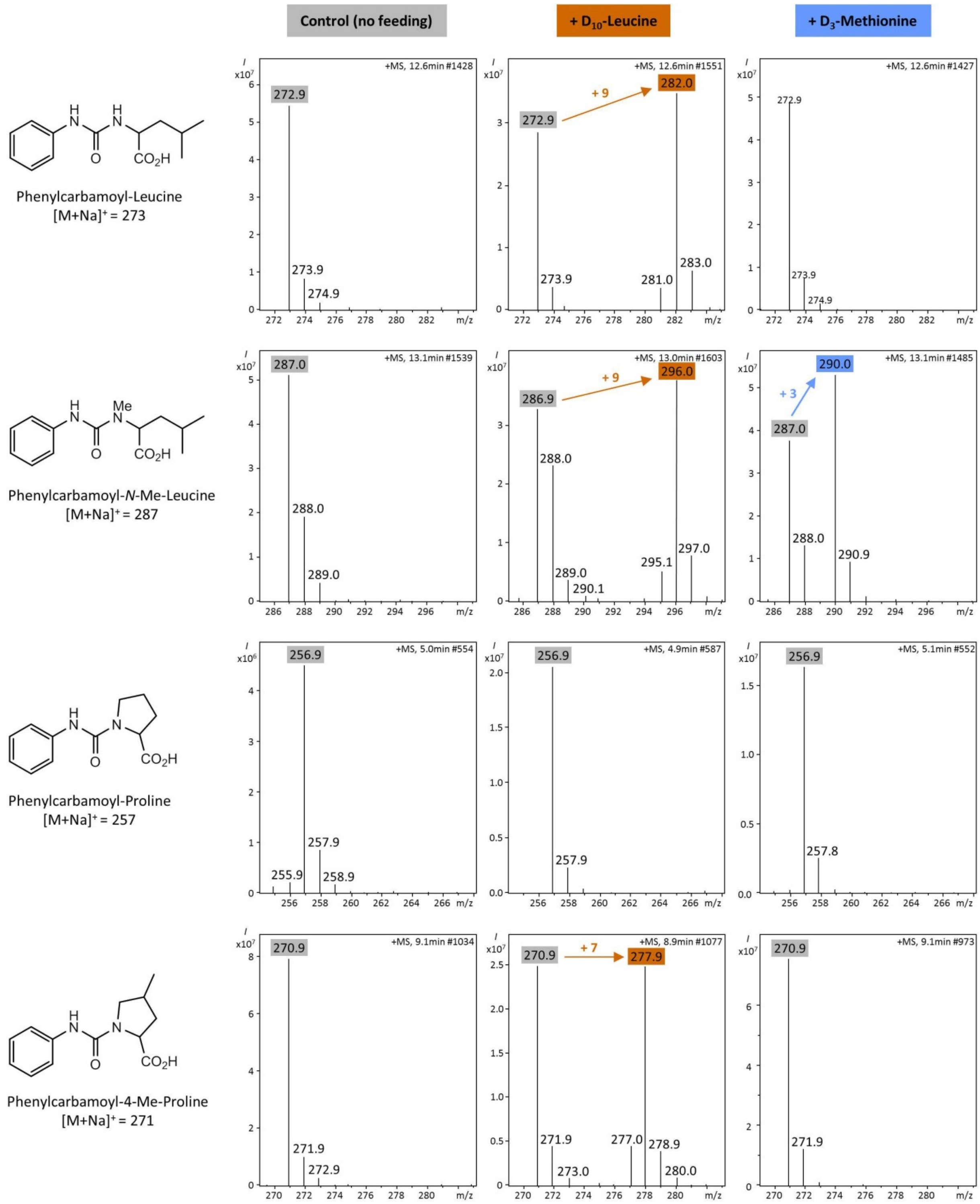

Figure S5. Feeding experiments to elucidate the biosynthetic origin of the methyl-proline units from griselimycin.

D_{10}-leucine and D_3-methionine was fed to cultures of the griselimycin producer; small amounts of griselimycin were purified, hydrolyzed and after derivatization the amino acid mixture was analyzed by HPLC-MS. The isotope pattern of derivatized leucine (1st row), *N*-Me-leucine (2nd row), proline (3rd row) and 4-Me-proline (4th row) are shown for each feeding experiment in comparison to the control (no feeding). Observed mass shifts attributed to the incorporation of deuterium-labeled precursors are indicated with arrows.

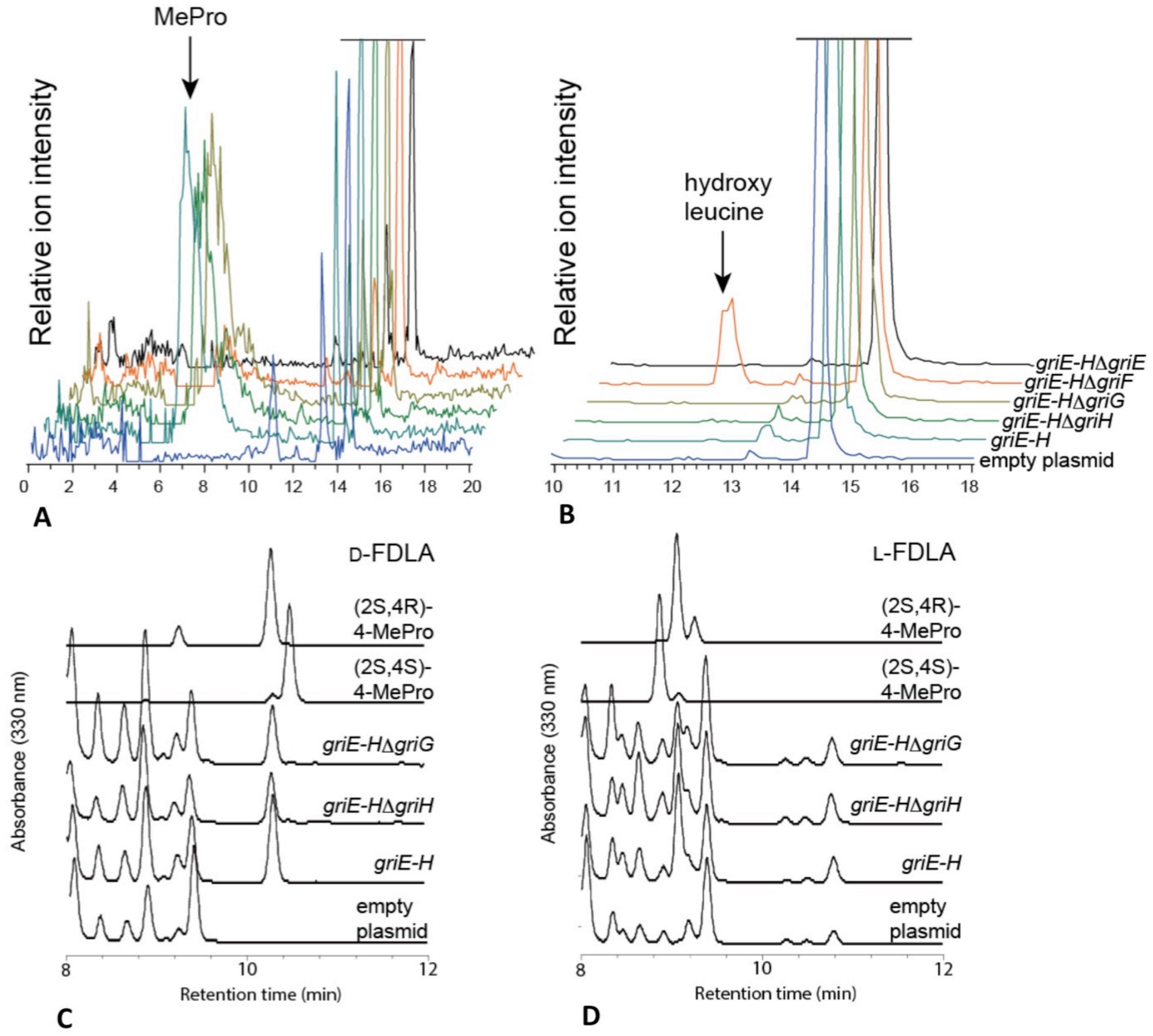

Figure S6. LC-MS Analysis of the 4-MePro produced by *Streptomyces lividans* harboring plasmids containing 4-MePro-biosynthesis genes.

(**A**) Extracted ion chromatogram (*m/z* 130) corresponding to MePro in positive ion mode. (**B**) Extracted ion chromatogram (*m/z* 148) corresponding to hydroxyleucine in positive ion mode. The stereochemistry was determined by Marfey's method via derivatization of the produced 4-MePro with 1-fluoro-2,4-dinitrophenyl-5-D-leucinamide (D-FDLA) (**C**) or 1-fluoro-2,4-dinitrophenyl-5-L-leucinamide (L-FDLA) (**D**). (2*S*,4*R*)-4-MePro and (2*S*,4*S*)-4-MePro: Pure reference compounds. The other lanes indicate cell extracts of *S. lividans* TK24 harboring different expression plasmids. *griE-H*: Plasmid contains the genes *griE-H* *griE-H*Δ*griE*: Plasmid contains the genes *griE-H* but not *griE. griE-H*Δ*griF*: Plasmid contains the genes *griE-H* but not *griF. griE-H*Δ*griG:* Plasmid contains the genes *griE-H* but not *griG*. *griE-H*Δ*griH:* Plasmid contains the genes *griE-H* but not *griH*. Empty plasmid: Cell extract of *S. lividans* TK24 harboring the empty control plasmid pUWL218. In all cases (except for the negative control) the production of the diastereomer (2*S*,4*R*)-4-MePro was observed suggesting that GriG and GriH do not have function for controlling stereochemistry. The plasmids used and the results obtained in this figure is summarized in table S8 and S9, respectively.

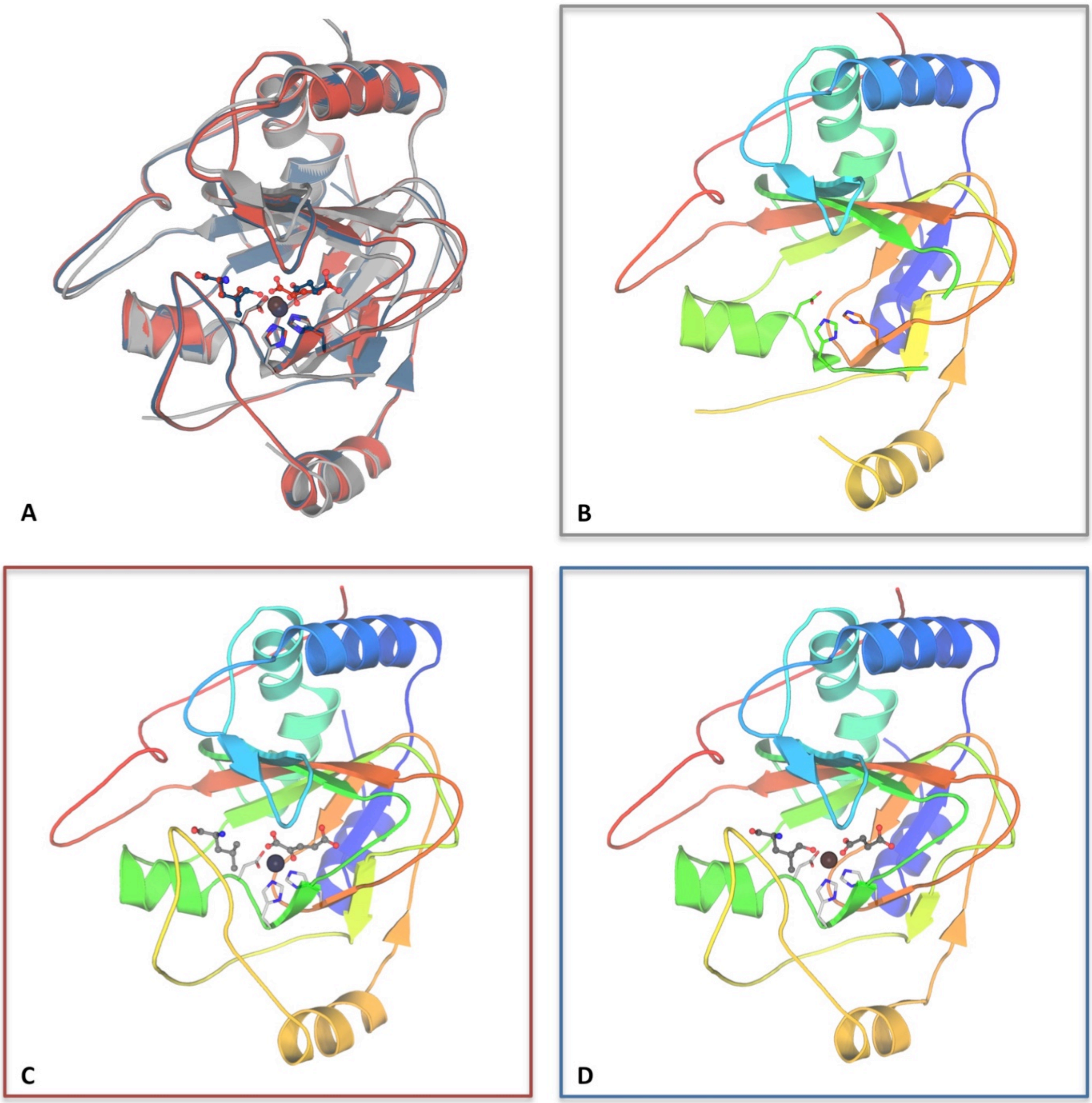

Figure S7. Overall structure of GriE.
(**A**) Superposition of the apo-enzyme (grey) with the Co^{2+}, α-ketoglutarate and L-leucine co-structure (red) and the Mn^{2+} containing product complex (blue). (**B**) Overview of the apo-structure colored from the N-terminus (blue) to the C-terminus (red). The metal-chelating residues are shown as sticks. Loop-regions involved in substrate binding were poorly defined in the electron density maps and are thus missing from the model. (**C**) Structural overview of the substrate complex colored from the N-terminus (blue) to the C-terminus (red). The substrates and the metal chelating residues are shown in grey. Co^{2+} is depicted as a sphere. (**D**) Structural overview of the product complex colored from the N-terminus (blue) to the C-terminus (red). The reaction products and the metal chelating residues are shown in grey. Mn^{2+} is depicted as a sphere.

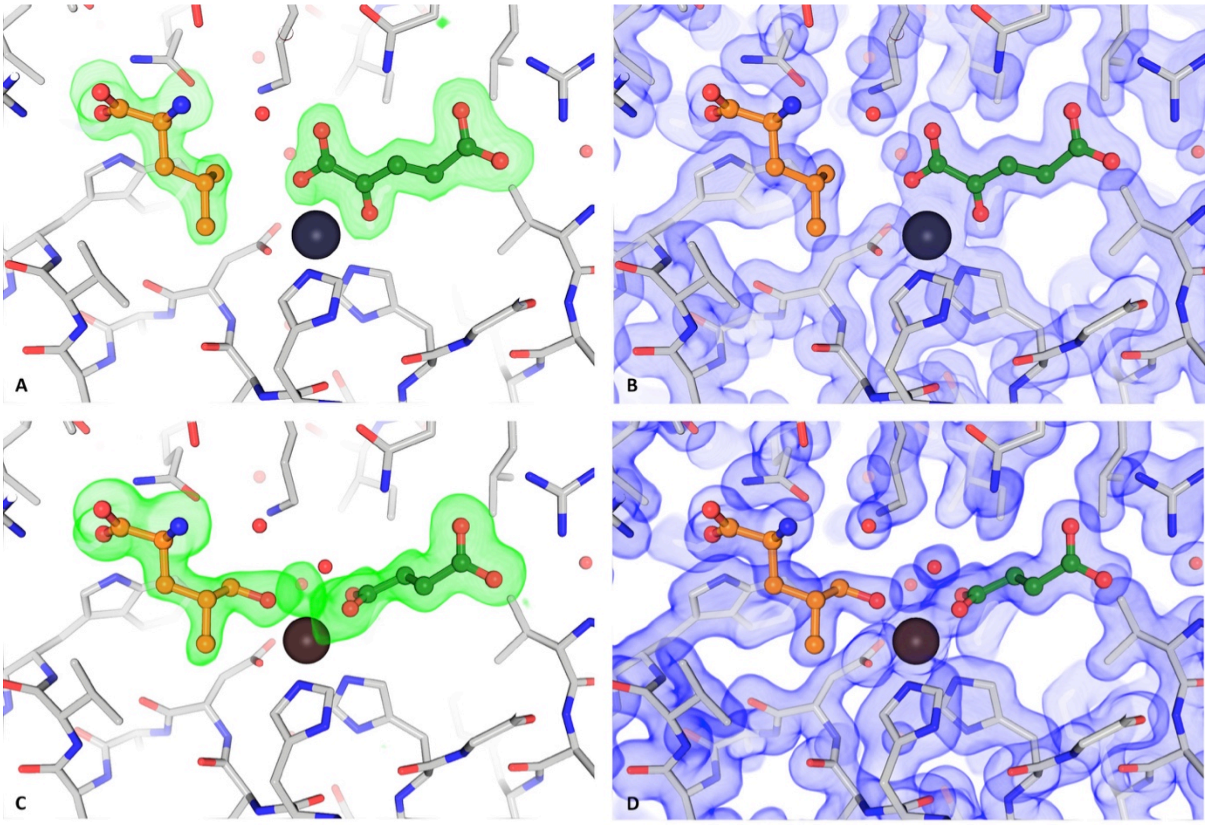

Figure S8. Electron density maps for the ligands bound in the GriE structures.
(**A**) Fit of L-leucine (orange) and α-ketoglutarate (green) into the positive mF_{obs}-DF_{calc} difference electron density map (contoured at σ = +3) after refinement of the structural model of the GriE substrate complex with omitted substrates. (**B**) $2mF_{obs}$-DF_{calc} electron density map in the active center of the GriE substrate complex (contoured at σ = +1) after refinement with the substrates included. (**C**) Fit of 5-hydroxyleucine (orange) and succinate (green) into the positive mF_{obs}-DF_{calc} difference electron density map (contoured at σ = +3) after refinement of the Mn^{2+} containing structural model of GriE against the dataset corresponding to the co-crystallization setup with Mn^{2+}, α-ketoglutarate and L-leucine. The hydroxyl-group of 5-hydroxyleucine is clearly defined in the electron density map. (**D**) $2mF_{obs}$-DF_{calc} electron density map in the active center of the GriE product complex (contoured at σ = +1) after refinement with the reaction products included. The shape of the electron density maps for 5-hydroxyleucine clearly identify this moiety as the (2*S*,4*R*)-diastereomer.

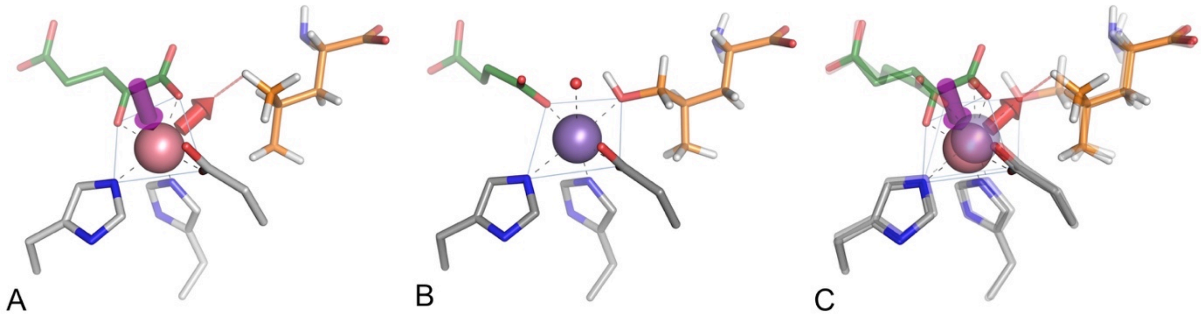

Figure S9. Active site geometry of the GriE-ligand complexes.
(**A**) Cobalt/substrate complex. Histidine 210, aspartate 112 and α-ketoglutarate (green) as bi-dentate ligand coordinate to the metal in a tetragonal plane (blue outline). While one of the axial positions is occupied by histidine 110, the other axial position is vacant in the crystal structure (purple arrow) and would allow for a dioxygen molecule to bind to the metal. In order to perform an attack on the substrate L-leucine (orange, shown with hydrogens), the reactive oxygen species formed during the decarboxylation of α-ketoglutarate would have to shift to an orthogonal binding site at the metal (red arrow). It could then

abstract a hydrogen (dashed red line) from $C_{\delta2}$ of leucine. (**B**) Manganese/product complex. A tetragonal plane (blue outline) of metal coordinating ligands is formed by histidine 210, aspartate 112, succinate (green) and the hydroxyl group of (2*S*,4*R*)-5-hydroxyleucine (orange, shown with hydrogens). The axial positions are occupied by histidine 110 and a water molecule (or carbon dioxide at a low partial occupancy). (**C**) Superimposition of the substrate and product complexes. Compared to α-ketoglutarate, succinate has rotated about 90° around its longitudinal axis, indicating the rearrangement of the metal coordination sphere during the reaction. The position of the reactive oxygen species required for performing the attack on the substrate L-leucine would then correspond to the position of the hydroxyl group of 5-hydroxyleucine in the product complex.

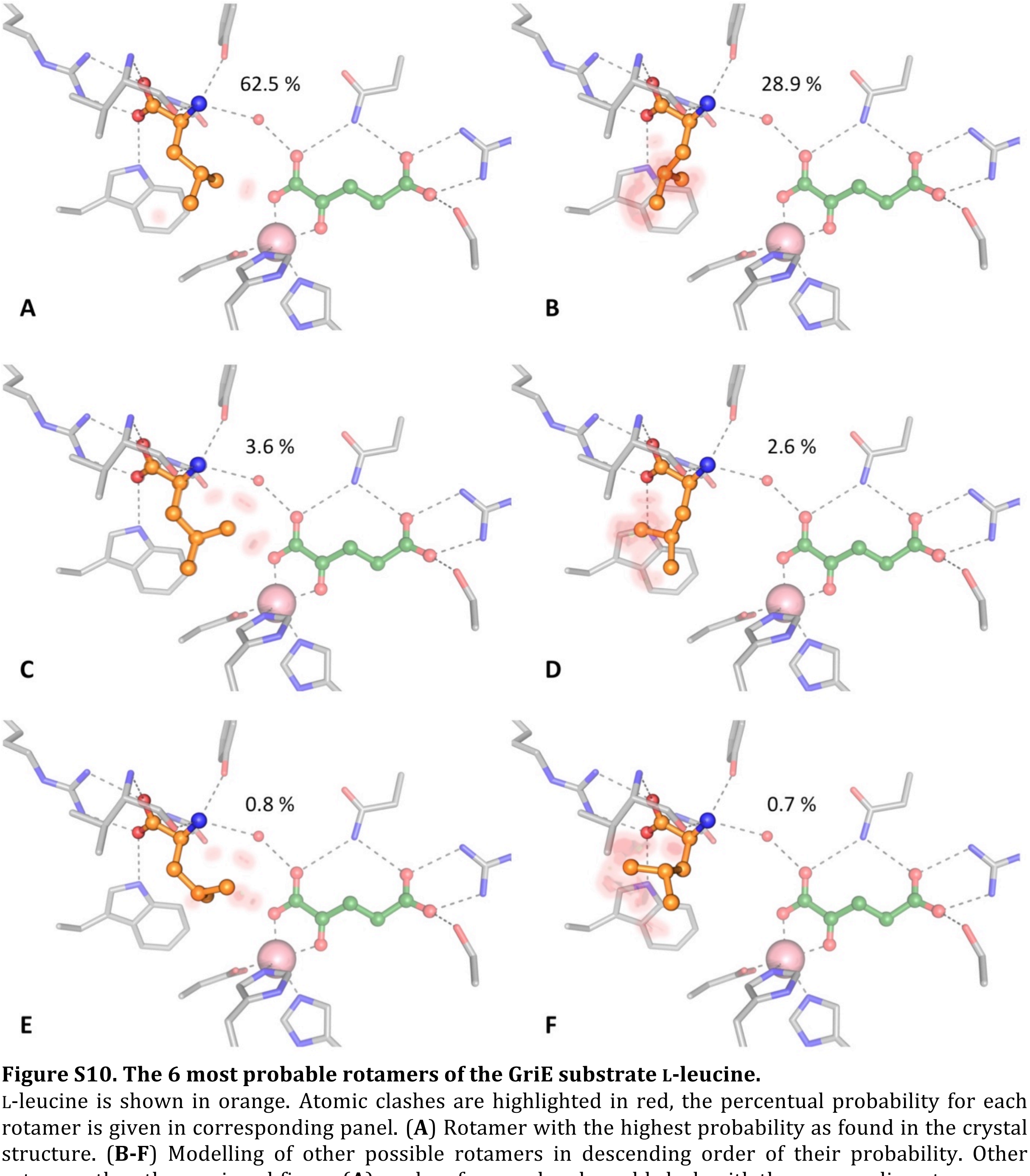

Figure S10. The 6 most probable rotamers of the GriE substrate L-leucine.
L-leucine is shown in orange. Atomic clashes are highlighted in red, the percentual probability for each rotamer is given in corresponding panel. (**A**) Rotamer with the highest probability as found in the crystal structure. (**B-F**) Modelling of other possible rotamers in descending order of their probability. Other rotamers than the one in subfigure (**A**) are less favoured and would clash with the surrounding atoms.

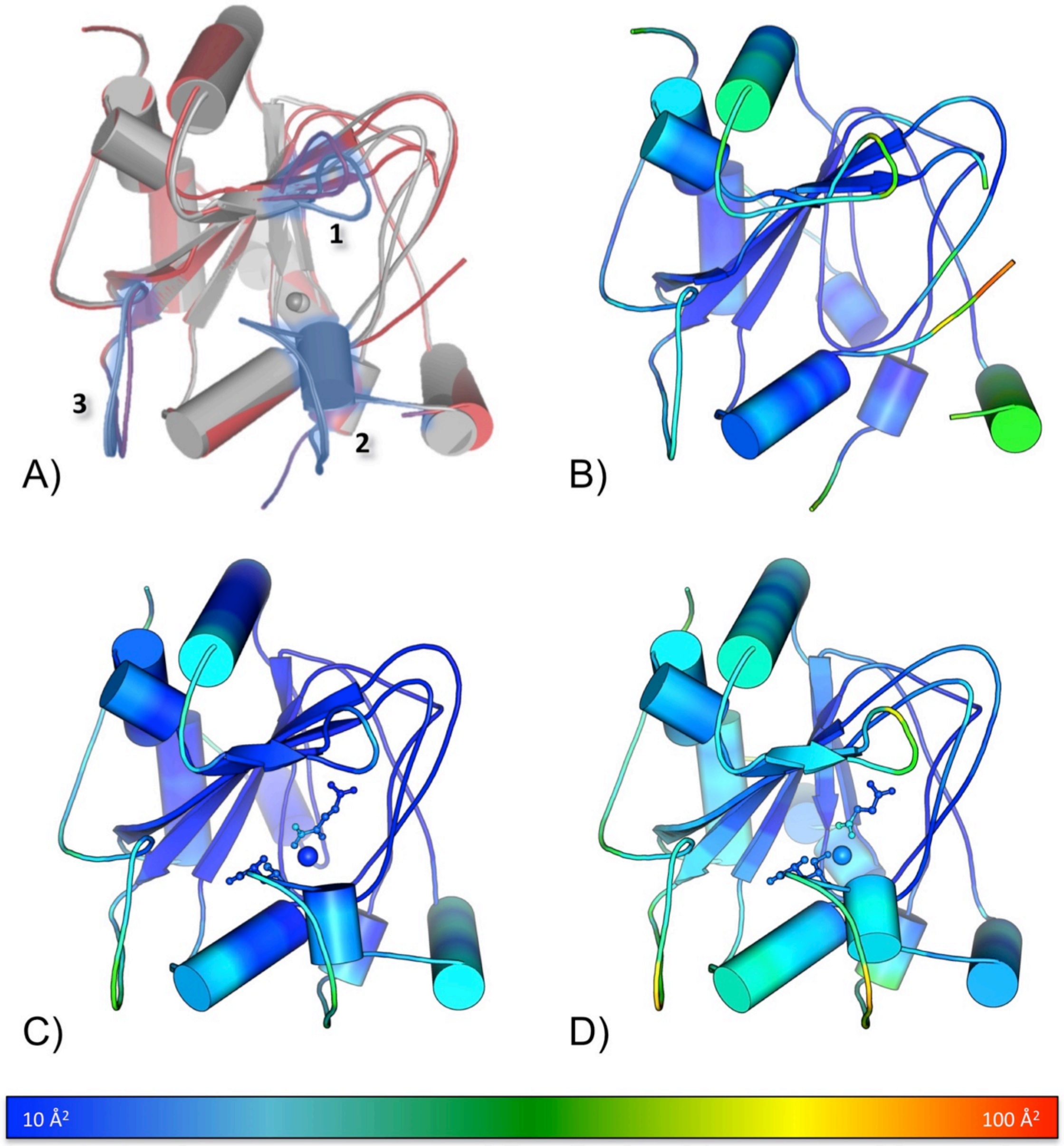

Figure S11. Conformational and B-factor analysis of the GriE crystal structures.
(**A**) Superposition of the three crystal structures. The apo structure is shown as red cartoon while the ligand complexed structures are shown in grey. For orientational purposes, the metal ions are shown as spheres. Loop regions that show conformational differences between the apo form and the ligand complexes are highlighted in blue. 1: The loop comprising the residues 48-57 seems to be shifted towards the active site in the ligand containing structures and thus seems to be involved in closing off the active site after substrate binding. 2: The residues 159-176 could not be modelled for the apo structure, but this region comprising residues 154-178 is well defined in the ligand complexes. Valine 170 from this region is involved in hydrogen bonding to (5-hydroxy-)leucine. 3: The loop comprising the residues 232-247 is tilted away from the active site in the ligand complexes. This can be assigned to arginine 242, which pushed out when forming a salt-bridge to the carboxylate of (5-hydroxy-)leucine. (**B**) Cartoon representation of the apo structure colored according to its crystallographic B-factors. The region of loop 2 comprising the residues 159-176 could not be modelled due to high disorder. Also glycine 104 within a β-strand of the jelly-roll core was not defined, probably because of increased flexibility of this area due to the missing metal ion. Loops 1 and 3 show elevated B-factors indicating their conformational flexibility. (**C**) Cartoon representation of the Co^{2+}/substrate complex colored according to its crystallographic B-factors. (**D**) Cartoon representation of the Mn^{2+}/product complex colored according to its crystallographic B-factors. In both ligand bound forms, the flexible loops 1-3 are well defined, but show slightly elevated B-factors indicating their conformational flexibility.
B-factors in sub-figures B-D are visualized from 10 $Å^2$ (blue) to 100 $Å^2$ (red).

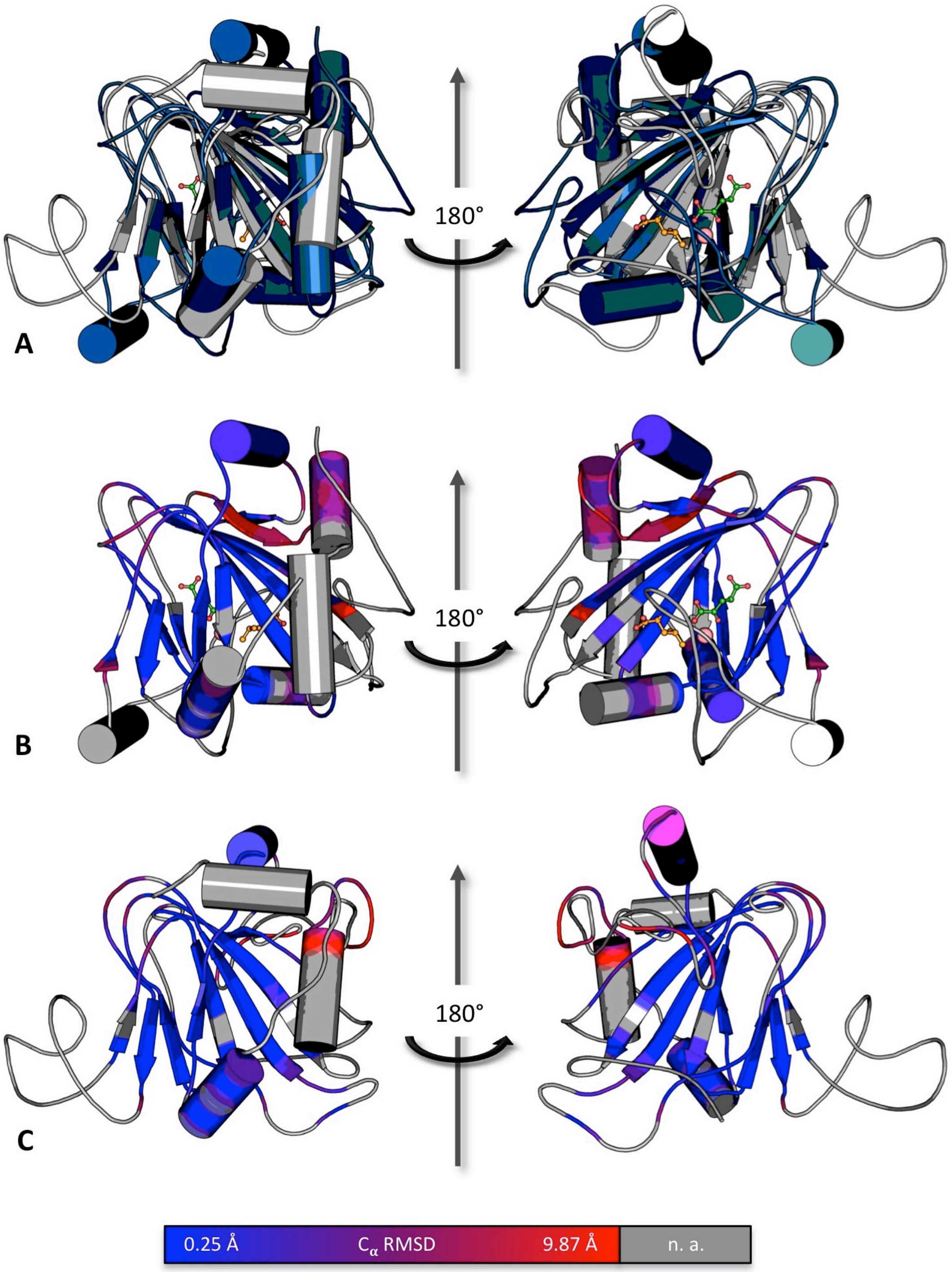

Figure S12. Structural comparison of GriE and a homology model of LdoA.
(**A**) Superposition of the crystal structure of the GriE substrate complex (blue cartoon, substrates and metal shown as sticks and spheres) and a homology model of LdoA from *Nostoc punctiforme* (grey). The homology model has been generated by the Phyre2[37] server with 84 % of the residues modelled with a confidence > 90 %. (**B**) Crystal structure of the GriE substrate complex with the cartoon representation colored according to the R.M.S.D.-values of the structural superposition of the C_α-carbons, ranging from 0.25 Å (blue) to 9.87 Å (red) with an average R.M.S.D. of the aligned $C\boldsymbol{\alpha}$-carbons of 2.62 Å. Areas not involved in the structural alignment are colored in grey. (**C**) Homology model of LdoA from *Nostoc punctiforme* colored according to subfigure (B). While the canonical jelly-roll core is highly conserved in both proteins, the regions involved in binding of the substrate L-leucine are not conserved among GriE and LdoA. This suggests that LdoA binds L-leucine with a different orientation to the metal than GriE, thus resulting in different stereochemistry of the reaction product

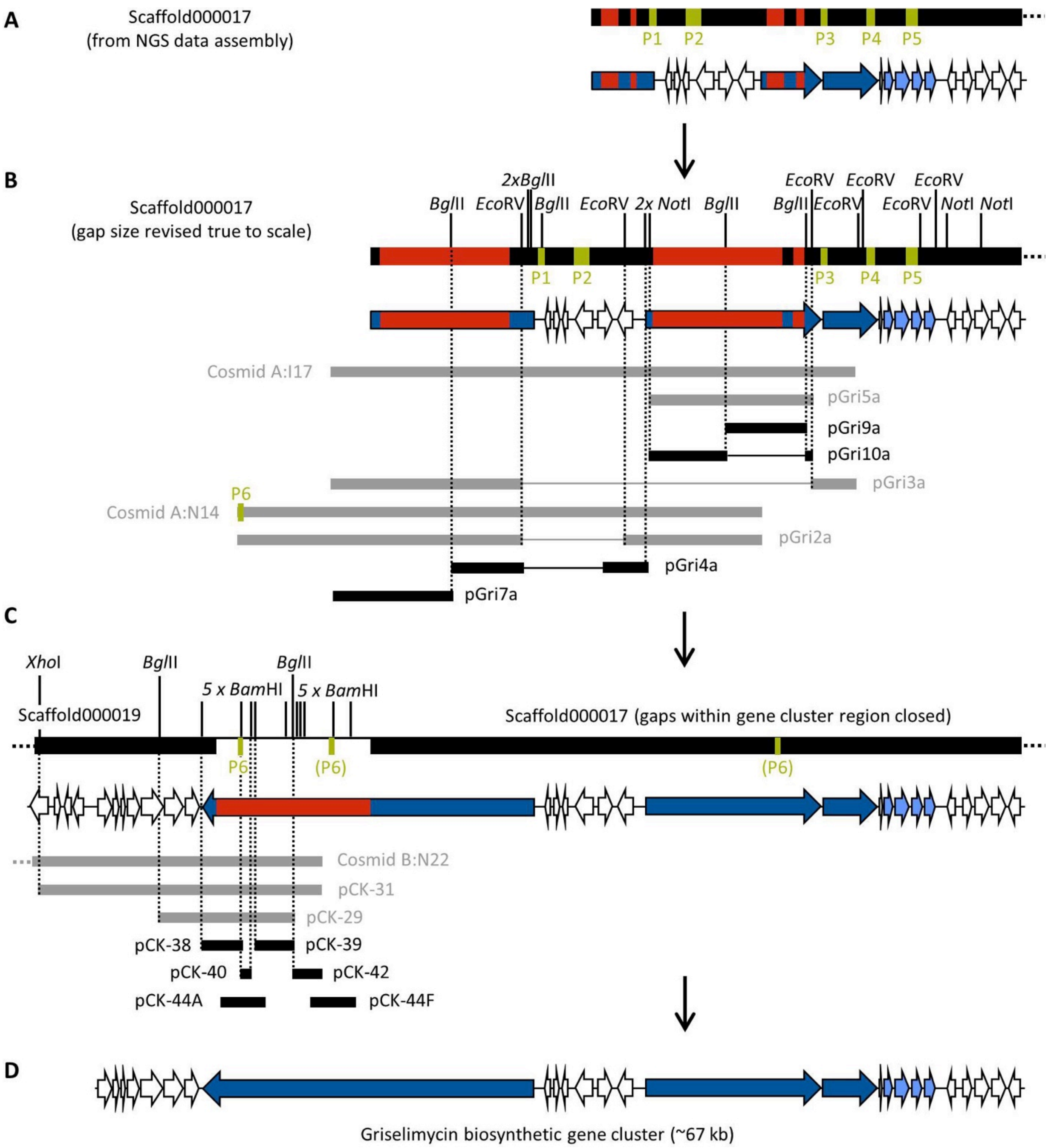

Figure S13. Decoding the griselimycin biosynthetic gene cluster sequence.
(**A**) Primary result from next-generation sequencing (NGS). Scaffold000017 harboring four gaps (in red) in the putative griselimycin gene cluster region was identified and used as template to design five probes (P1-P5, in green) for screening the *Streptomyces* DSM 40835 cosmid library. (**B**) Subcloning of unknown gene cluster fragments from cosmids A:I17 and A:N14. Plasmid inserts shown in black were completely sequenced to decipher the entire gene cluster sequence from Scaffold000017. (**C**) Extended subcloning approaches based on cosmid B:N22 identified via hybridization with probe P6 (cosmid end-sequencing data established the connection to Scaffold000019). Plasmid inserts shown in black were completely sequenced. (**D**) Map of the griselimycin biosynthetic gene cluster after elucidation of the complete sequence. In total, 30 kb of unknown DNA (gaps shown in red) within the NRPS encoding genes (shown in dark blue) was subcloned and sequenced.

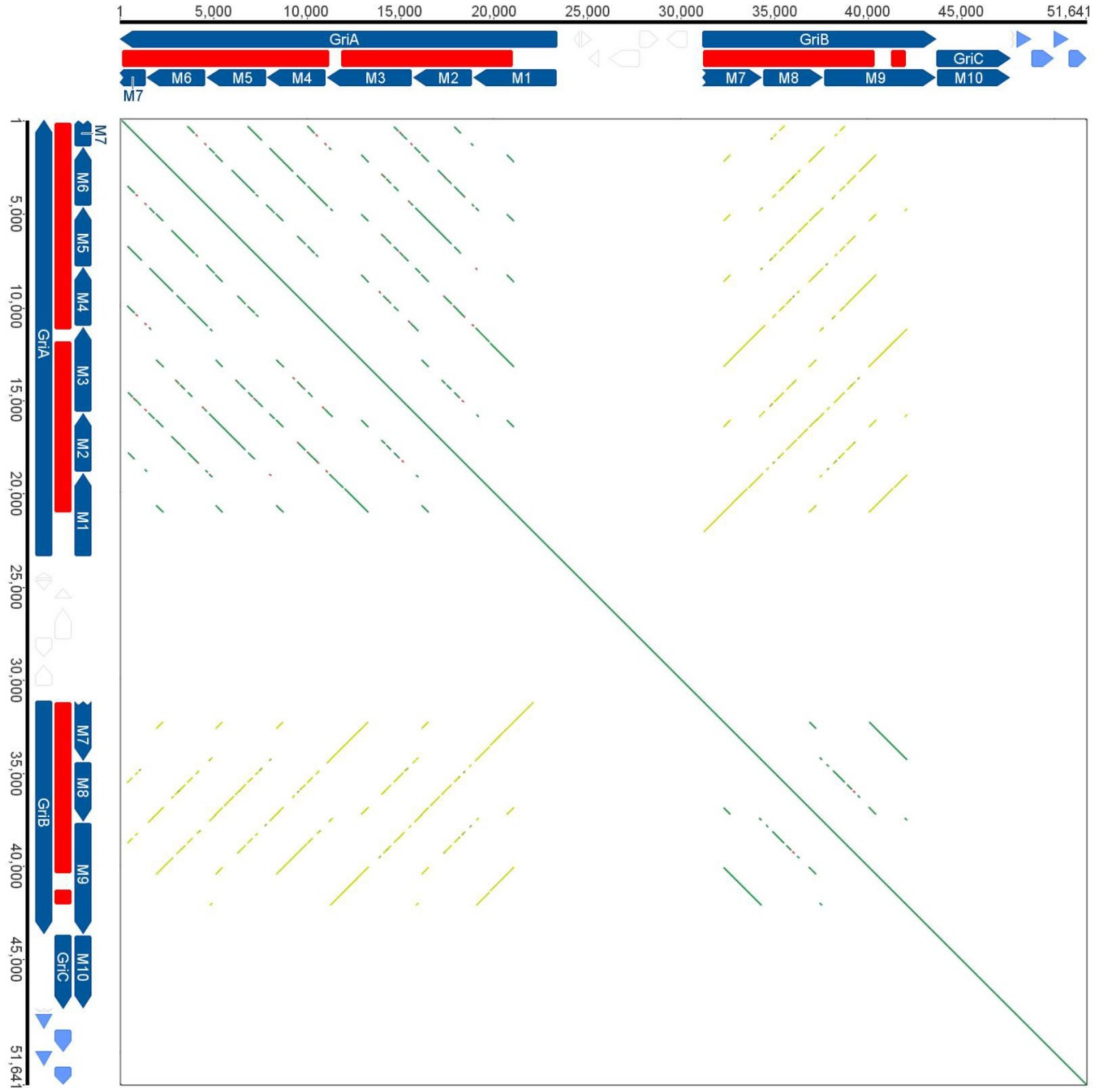

Figure S14. Dotplot of the griA-griH griselimycin biosynthetic gene cluster region.
The encoded NRPS megasynthetase GriABC harboring 10 modules (M1-M10) is illustrated in dark blue, the putative methyl-proline biosynthesis gene set (*griE-H*) is shown in light blue. The sequence gaps within *griA* and *griB* from the NGS data assembly are highlighted in red. Dotplot analysis was carried out with the Geneious 8.1.3 software based on the EMBOSS 6.5.7 tool dotup.

```
                 **   *  * * *    *        **
A2          LWHTFDVSAQESFAAQGGEHNHYGPTETHAVTAY   DVQFAGHAVK   (2S,4R)Me-Pro
A5          LWHTFDVSAQESFAAQGGEHNHYGPTETHAVTAY   DVQFAGHAVK   (2S,4R)Me-Pro
A8          LWHTFDVSAQESFAAQAGEHNHYGPAETHVVTAH   DVQFAAHVVK   Pro/(2S,4R)Me-Pro
NcpB-A3     LWHAFDVSFQETFLITAGEHNHYGPSESHVATSF   DVQFIAHVAK   (2S,4S)Me-Pro
NosA-A3     LWHAFDVSFQETFLITAGEHNHYGPSESHLATSF   DVQFIAHLAK   (2S,4S)Me-Pro
NosD-A2     LWQAFDVSFQETFVITAGEHNHYGPSESHVIITF   DVQFIAHVIK   Pro
```

Figure S15. Comparison specificity-conferring residues of (methyl)proline specific adenylation (A) domains of the griselimycin (A2, A5 and A8), nostopeptolide (NosA-A3 and NosD-A2) and nostocyclopeptide (NcpB-A3) megasynthetase.
The two sequence columns represent the 8Å signature (a set of 34 active site residues as defined by Rausch *et al.*[14]) and the Stachelhaus code (ten specificity-conferring residues defined by Stachelhaus *et al.*[15] and Challis *et al.*[16]; they correspond to position 235, 236, 239, 278, 299, 301, 322, 330, 331, 517 of the gramicidin S synthetase A domain (GrsA, PDB-ID: 1AMU);[72] nine of these ten residues are part of the 8Å signature as labeled with asterisks). The 8Å signature/Stachelhaus code for each A domain was retrieved via the NRPSpredictor2 analysis tool.[73] The actual incorporated substrate by the respective NRPS module is shown rightmost. Sequences from the nostopeptolide/nostocyclopeptide megasynthetase[12, 13] A domains were retrieved from GenBank: AAF15891.2 (NosA-A3: aa 2678-3167), AAF17281.1 (NosD-A2: aa 1572-2042), AAO23334.1 (NcpB-A3: aa 2695-3228).

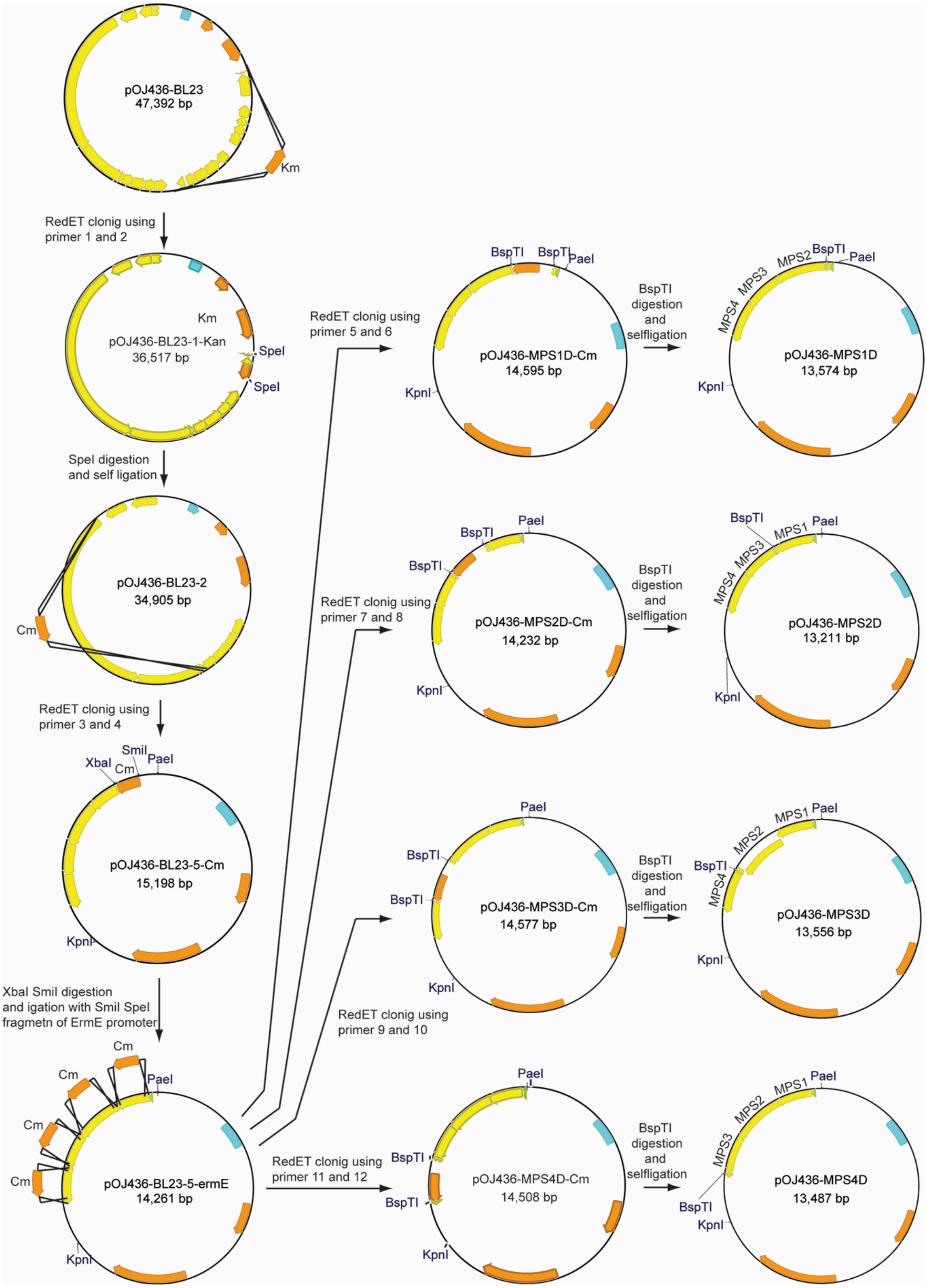

Figure S16. Construction of cosmids for heterologous expression of the 4-MePro biosynthesis geneset.

The designation *mps1-4* for the genes of the 4-MePro-biosynthesis sub-operon corresponds to the genes *griE-H*. The cosmids were constructed using RedET cloning (GB2005red, Gene Bridges GmbH, Heidelberg, Germany). Primers used for this experiment is summarized in Table S12.

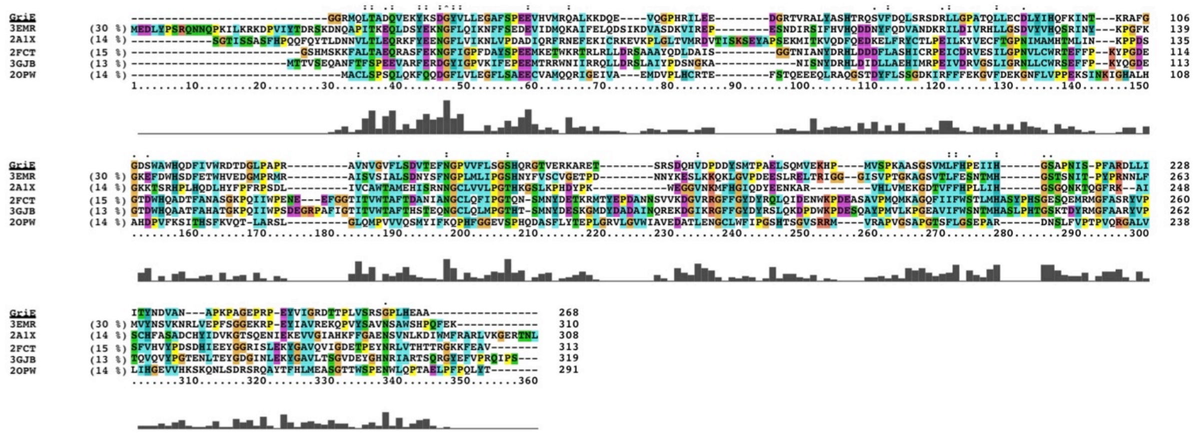

Figure S17. Alignment of the five closest homologues in the PDB, which were used for model generation for molecular replacement to the GriE 1-265 sequence.
The sequence identities of the aligned sequences to GriE 1-265 are given in parentheses behind the PDB identifiers. 3EMR: Ectoine hydroxylase EctD from *Salibacillus salexigens*[74]. 2A1X: Human phytanoyl-coa 2-hydroxylase[75]. 2FCT: Nonheme iron halogenase SyrB2 from syringomycin biosynthesis[76]. 3GJB: Nonheme iron halogenase CytC3[77]. 2OPW: Human phytanoyl-CoA dioxygenase PHYHD1 (Zhang *et al.*, unpublished). The alignment was generated using Clustax X 2.1[70, 71].

IV. Supplementary Tables

Table S1. Genes and encoded proteins of the griselimycin biosynthetic gene cluster.

No.	Gene name	Gene length [nt]	Protein name	Protein length [aa]	Putative function
1	*orf1*	1,269	Orf1	412	XRE family transcriptional regulator
2	*orf2*	441	Orf2	146	Hypothetical protein
3	*orf3*	195	Orf3	64	Hypothetical protein
4	*orf4*	1,005	Orf4	334	Hypothetical protein
5	*orf5*	1,521	Orf5	506	Amino acid permease
6	*orf6*	1,440	Orf6	479	Glutamine synthetase
7	*orf7*	894	Orf7	297	Phosphotransferase enzyme family
8	*griA*	23,385	GriA	7,794	Nonribosomal peptide synthetase‡ (C-A-MT-CP-C-A-CP-C-A-MT-CP-C-A-CP-C-A-CP-C-A-CP-C)
9	*int1*	333	Int1	110	Integrase catalytic region
10	*tnp1*	540	Tnp1	179	Transposase
11	*int2*	561	Int2	186	Integrase catalytic region
12	*tnp2*	1,653	Tnp2	550	Transposase
13	*tnp3*	1,032	Tnp3	343	Transposase
14	*griR*	1,122	GriR	373	DNA polymerase III, beta subunit
15	*griB*	12,507	GriB	4,168	Nonribosomal peptide synthetase‡ (A-MT-CP-C-A-CP-C-A-MT-CP-E)
16	*griC*	3,975	GriC	1,324	Nonribosomal peptide synthetase‡ (C-A-CP-TE)
17	*griD*	219	GriD	72	MbtH-like protein
18	*griE*	813	GriE	270	Leucine hydroxylase
19	*griF*	1,176	GriF	391	Alcohol dehydrogenase
20	*griG*	801	GriG	266	Alpha/beta hydrolase
21	*griH*	900	GriH	299	Oxidoreductase
22	*orf8*	441	Orf8	146	Pyridoxamine 5‘-phosphate oxidase family protein
23	*orf9*	651	Orf9	216	Hypothetical protein
24	*orf10*	1,116	Orf10	371	Hypothetical protein
25	*orf11*	1,197	Orf11	398	Amidohydrolase family protein
26	*orf12*	903	Orf12	300	XRE family transcriptional regulator

Nt = nucleotides, aa = amino acids
‡ Abbreviations of catalytic nonribosomal peptide synthetase (NRPS) domains: C = condensation domain, A = adenylation domain, MT = methylation domain, CP = carrier protein domain, E = epimerization domain, TE = thioesterase domain.

Table S2. Substrate specificity analysis of the 10 adenylation (A) domains from the griselimycin megasynthetase.

	Specificity-conferring code [a]										Predicted substrate [b]				Incorpo-rated [c]
	235	236	239	278	299	301	322	330	331	517					
**A1**	D	F	F	C	V	G	I	V	H	K	Thr	Thr	Thr	**Thr**	Val
**A2**	D	V	Q	F	A	G	H	A	V	K	Pro	Pro	Pro	**Pro**	MePro
**A3**	D	F	W	N	V	G	M	V	H	K	Thr	Thr	Thr	**Thr**	Thr
**A4**	D	A	L	L	L	G	A	V	V	K	‘asc‘	Tyr	Trp	**-**	Leu
**A5**	D	V	Q	F	A	G	H	A	V	K	Pro	Pro	Pro	**Pro**	MePro
**A6**	D	A	L	L	L	G	A	V	V	K	‘asc‘	Tyr	Trp	**-**	Leu
**A7**	D	F	F	C	V	G	I	V	H	K	Thr	Thr	Thr	**Thr**	Val
**A8**	D	V	Q	F	A	A	H	V	V	K	Pro	Pro	Pro	**Pro**	Pro/MePro
**A9**	D	A	L	L	L	G	A	V	V	K	‘asc‘	Tyr	Trp	**-**	Leu
**A10**	D	I	L	Q	L	G	V	I	W	K	Gly	Gly	Gly	**Gly**	Gly

[a] Residues defined according to Stachelhaus *et al.*[15] and Challis *et al.*[16]; residue numbering corresponds to gramicidin S synthetase A domain numbering (GrsA, PDB-ID: 1AMU)[72]

[b] Retrieved from reports of the applied antiSMASH 3.0 gene cluster analysis,[6] including substrate predictions based on the NRPSPredictor2 method[73] (1st column), Stachelhaus code[15] (2nd column), method of Minowa *et al.*[78] (3rd column), and the consensus of the three approaches highlighted in bold (4th column; ‘-‘ indicates no consensus). In some cases, no single substrates but a group of several amino acids with similar physico-chemical properties were predicted by NRPSPredictor2 as indicated by ‘asc’ = aromatic side chain amino acids (phe, trp, phg, tyr, bht).

[c] Amino acids incorporated in the actual (methyl)griselimycin structure.

Table S3. GM and MGM production in cultures of *Streptomyces* DSM 40835 fed with (2*S*,4*R*)-4-methyl-proline.

(2*S*,4*R*)-4-methyl-proline (µg/ml)	Day 1		Day 2		Day 3		Day 4	
	GM (mg/l)	MGM (mg/l)	GM (mg/l)	MGM (mg/l)	GM (mg/l)	MGM (mg/l)	GM (mg/l)	MGM (mg/l)
0	3.8	0.0	106.9	4.2	180.5	5.6	185.0	5.0
	3.7	0.4	131.1	3.8	177.0	5.0	166.5	4.4
	4.3	0.0	140.0	5.2	172.4	5.0	163.6	4.2
20	3.9	0.2	90.0	8.4	151.2	20.6	147.2	18.6
	3.7	0.5	77.8	11.2	141.4	27.7	155.7	23.4
	3.7	0.3	94.5	12.4	155.4	20.9	151.0	18.6
200	4.1	0.0	52.5	14.8	70.1	51.9	98.1	41.7
	3.5	0.0	58.2	11.6	75.9	38.9	105.5	43.1
	4.2	0.2	59.9	14.2	70.5	53.0	94.6	46.2

Table S4. X-ray data collection & refinement statistics

**Structure**	GriE: apo	GriE: Co^{2+} & substrates	GriE: Mn^{2+} & products
PDB-ID:	5NCH	5NCI	5NCJ
**Data collection**			
Beamline	in-house Cu-anode	SLS X06DA (PXIII)	SLS X06DA (PXIII)
Wavelength (Å)	1.54187	1.00003	1.00003
Space group	$P2_1$	C2	C2
Cell dimensions			
a, b, c (Å)	44.37, 69.84, 98.38	148.72, 56.06, 73.53	148.74, 56.06, 73.80
α, β, γ (°)	90.00, 90.18, 90.00	90.00, 111.95, 90.00	90.00, 112.46, 90.00
Resolution (Å)[a]	98.39 - 1.82 (1.85 – 1.82)	68.97 – 1.76 (1.79 – 1.76)	68.73 - 1.53 (1.56 – 1.53)
R_{merge} (%)[a]	8.1 (71.1)	9.4 (85.2)	9.3 (86.7)
R_{pim} (%)[a]	5.0 (45.9)	3.9 (34.2)	3.8 (36.2)
$I/\sigma I$[a]	11.4 (2.2)	14.0 (2.2)	11.0 (2.1)
Completeness (%)[a]	86.3 (84.0)	99.1 (98.1)	94.2 (99.5)
Redundancy[a]	3.5 (3.4)	6.9 (7.1)	6.7 (6.6)
$CC_{1/2}$[32] (%)[a]	99.7 (78.6)	99.8 (73.4)	99.5 (75.7)
**Refinement**			
Resolution (Å)	1.82	1.76	1.53
No. reflections	46611	55867	80165
R_{work}/R_{free} (%)	17.22 / 21.28	13.97 / 16.93	15.54 / 17.34
No. atoms	4992	5174	5310
Protein	4474	4612	4648
Ligand/ion	0	41	38
Water	518	521	624
B-factors (Å^2)	26.67	27.41	27.99
Protein	25.71	26.33	26.81
Ligand/ion	0.00	21.71	20.79
Water	34.91	37.45	37.19
R.m.s deviations			
Bond lengths (Å)	0.007	0.011	0.007
Bond angles (º)	0.810	1.038	0.923
Ramachandran statistics (%)			
Favored	98.77	99.62	99.42
Allowed	1.23	0.38	0.58
Outliers	0.00	0.00	0.00

[a]Values for the highest resolution shell are shown in parentheses.

Table S5. Probes used to screen the *Streptomyces* DSM 40835 cosmid library.

Name	Description
Probe 1	505 bp fragment generated with oligonucleotides Sca17_17481F (5'-GATAACGCTGTTCGTCATTCCAG-3') / Sca17_17780R (5'-TCCTCAAAGACGACTGCCGTC-3')
Probe 2	1109 bp fragment generated with oligonucleotides Gri11 (5'-CTCACGCAACTTCACATAGC-3') / Gri12 (5'-CTGGAACTACACCTTGGCTC-3')
Probe 3	448 bp fragment generated with oligonucleotides Sca125do (5'-GCTCTAGAAGCTCTTCCGCCAGGAT-3') / Sca125up (5'-CGGGATCCATGGAGGGCATCGGAAT-3')
Probe 4	540 bp fragment generated with oligonucleotides Gri5 (5'-GCCAGTCTACGCGATTCAAG-3') / Gri6 (5'-GTCAGTGTCGACCTGAAGAG-3')
Probe 5	802 bp fragment generated with oligonucleotides Gri9 (5'-CGATTTGGTCATCGAGTGTG-3') / Gri10 (5'-CATCGATGAGATCGATCTCG-3')
Probe 6	223 bp fragment generated with oligonucleotides Gri21 (5'-GTCAGACCAGCACGGTTC-3') / Gri22 (5'-GCAGGCGCTTCATCAACG-3')

Table S6. Plasmids constructed to decipher the griselimycin biosynthetic gene cluster sequence.

Plasmid	Vector backbone/ Selection marker	Construction
pGri2a	pOJ436 / $Apra^R$	*Eco*RV restriction of cosmid A:N14, gel purification of the largest obtained fragment (ca. 40 kb) and religation
pGri3a	pOJ436 / $Apra^R$	*Eco*RV restriction of cosmid A:I17, gel purification of largest obtained fragment (ca. 26 kb) and religation
pGri4a	pBluescript II KS / Amp^R	Subcloning of the ~ 6.7 kb *Not*I/*Bgl*II fragment from pGri2a into pBluescript II KS linearized with *Not*I/*Bam*HI
pGri5a	pBluescript II KS / Amp^R	Subcloning of the 11.5 kb *Not*I/*Eco*RV fragment from cosmid A:I17 into pBluescript II KS linearized with *Not*I/*Eco*RV
pGri7a	pBluescript II KS / Amp^R	Subcloning of the 8.5 kb *Spe*I/*Bgl*II fragment from pGri3a into pBluescript II KS linearized with *Spe*I/*Bam*HI
pGri9a	pBluescript II KS / Amp^R	Subcloning of the 5.7 kb *Bgl*II fragment from pGri5a into pBluescript II KS linearized with *Bam*HI
pGri10a	pBluescript II KS / Amp^R	*Bgl*II restriction of pGri5a, gel purification of the largest obtained fragment (ca. 9 kb) and religation
pCK-31	pBluescript SK(+) / Amp^R	Subcloning of the 25 kb *Xho*I fragment from cosmid B:N22 into pBluescript SK(+) linearized with *Xho*I
pCK-29	pBluescript SK(+) / Amp^R	Subcloning of the 9.3 kb *Kpn*I/*Bgl*II fragment from cosmid B:N22 into pBluescript SK(+) linearized with *Bam*HI

pCK-38	pBluescript SK(+) / Amp^R	Subcloning of the 2.9 kb *Bam*HI fragment from pCK-29 into pBluescript SK(+) linearized with *Bam*HI
pCK-39	pBluescript SK(+) / Amp^R	Subcloning of the 2.6 kb *Bam*HI fragment from pCK-29 into pBluescript SK(+) linearized with *Bam*HI
pCK-40	pBluescript SK(+) / Amp^R	Subcloning of the 0.7 kb *Bam*HI fragment from pCK-29 into pBluescript SK(+) linearized with *Bam*HI
pCK-42	pBluescript SK(+) / Amp^R	Subcloning of the 7.8 kb *Bam*HI/*Xho*I fragment from pCK-31 into pBluescript SK(+) linearized with *Bgl*II/*Xho*I
pCK-44A	pBluescript SK(+) / Amp^R	Subcloning of the *Xba*I hydrolyzed PCR product generated with primers C4_2_for (5'-GC**TCTAGA**GGT GAGGTGTTCGAGTTCAGC-3') and C_B:N22_2_rev (5'-GC**TCTAGA**ATTTGAAGTACAGGGAGTCG-3') from chromosomal DNA of *Streptomyces* DSM 40835 into pBluescript SK(+) linearized with *Xba*I
pCK-44F	pBluescript SK(+) / Amp^R	Subcloning of the *Xba*I hydrolyzed PCR product generated with primers C4_2_for and C_B:N22_2_rev (see pCK-44A) from cosmid A:I4 into pBluescript SK(+) linearized with *XbaI*

Table S7. Construction of plasmids for inactivation of the griselimycin biosynthesis pathway.

Plasmid	Vector backbone/ Selection marker	Construction‡
pNRPS-1 (= pCK-8b)	pBluescript SK+ Amp^R	~4.1 kb *griA* fragment generated by PCR with oligonucleotides Nrps-1_for (5'-GC**TCTAGA**GTCATGCAGATCAACTCG-3') and Nrps-1_rev (5'-GC**TCTAGA**TGGTCACGGACCTGTG-3'), hydrolyzed with *Xba*I for subcloning into pBluescript SK+ linearized with the same enzyme
pNRPS-3 (= pCK-2a)	pBluescript SK+ Amp^R	~4.0 kb *griC* fragment generated by PCR with oligonucleotides Nrps-3_for (5'-GC**TCTAGA**ATGGAGGGCATCGGAAT-3') and Nrps-3_rev (5'-GC**TCTAGA**AGCTCTTCCGCCAGGAT-3'), hydrolyzed with *Xba*I for subcloning into pBluescript SK+ linearized with the same enzyme
pMPS (= pCK-12)	pBluescript SK+ Amp^R	~5.5 kb *mps* fragment reconstituted from two PCR products in pBluescript SK(+): the first fragment was generated with oligonucleotides Mps_for1 (5'-CGG**GGTACC**CATCTGGTCCGTACCAT-3') and Mps_rev1 (5'-TCGCGTCGTTTACCTCA-3') and hydrolyzed with *Kpn*I/*Xho*I for ligation into pBluescript SK(+) linearized with the same enzymes. The resulting plasmid (pCK-11) was linearized with *Xho*I/*Xba*I to subclone a second fragment generated with oligonucleotides Mps_for2 (5'-GCCTTCACCGGAGCTCA-3') and Mps_rev2 (5'-GC**TCTAGA**TCCTGAACTCTCGCATA-3') and hydrolyzed with *Xho*I/*Xba*I.
pNRPS-1 -apra-	pBluescript SK+ Amp^R/Apra^R	Amplification of the *apraR-oriT* cassette from pCK-1 using the oligonucleotides Nrps-1_for2

oriT (= pCK-8bRedET)		(5'-TGAGGTGTGTGCTGCGTCTGTTCCGGGGGCGACTGCGGTATTCCGGGGATCCGTCGACC-3') and Nrps-1_rev2 (5'-TGACACGTCGTGGCGTGCACCGTCGTCTCCGTCGGCCCGTGTAGGCTGGAGCTGCTTC-3'); the obtained PCR product was used to modify pNRPS-1 via Red/ET recombination
pNRPS-3 -apra-oriT (= pCK-2aRedET)	pBluescript SK+ Amp^R/$Apra^R$	Amplification of the *apraR-oriT* cassette from pCK-1 using the oligonucleotides DelSca125_for (5'-TCCGGATGCGACGGCAGTCGTCTTTGAGGACACGAGCGTATTCCGGGGATCCGTCGACC-3') and DelSca125_rev (5'-AGCATCACGTCGAACGCATTGTCTGCGGTGTCCATGTCTTGTAGGCTGGAGCTGCTTC-3'); the obtained PCR product was used to modify pNRPS-3 via Red/ET recombination
pMPS -apra-oriT (= pCK-12RedET)	pBluescript SK+ Amp^R/$Apra^R$	Amplification of the *apraR-oriT* cassette from pCK-1 using the oligonucleotides DelMPro2_for (5'-ATGAGCGATAGGTCTCCGTCTGTGGTAACCCCTGGTGTGATTCCGGGGATCCGTCGACC-3) and DelMpro2_rev (5'-TCAGGTATCGACCGTGGGAAGGTGCCGGCTTTCCAGTACTGTAGGCTGGAGCTGCTTC-3'); the obtained PCR product was used to modify pMPS via Red/ET recombination

‡Restriction sites used for cloning in bold, homology arms used for Red/ET recombination underlined.

Table S8. Plasmids for the heterlorogous expression of the MePro biosynthetic genes.

	originally from	*griE*	*griF*	*griG*	*griH*
pUWL-MPSs	pUWL218	Yes	Yes	Yes	Yes
pUWL-griE_D	pUWL218	no	Yes	Yes	Yes
pUWL-griF_D	pUWL218	Yes	no	Yes	Yes
pUWL-griG_D	pUWL218	Yes	Yes	no	Yes
pUWL-griH_D	pUWL218	Yes	Yes	Yes	no

Table S9. Heterologous expression of MPS genes *in S. lividans* TK24.

	methyl-proline	hydroxyleucine	Streochemistry of methyl proline[a]
pUWL-MPSs	Yes	no	(2*S*,4*R*)
pUWL-griE_D	no	no	-
pUWL-griF_D	no	yes	-
pUWL-griG_D	Yes	no	(2*S*,4*R*)
pUWL-griH_D	Yes	no	(2*S*,4*R*)

[a]Determined by using Marfey's method and comparing with the standard samples.

Table S10. Kinetic parameters determined for GriF.

V_{max} (nM/min)	22 ± 2
K_m (μM)	2.6 ± 1.7
k_{cat} (1/min)	0.12 ± 0.02
k_{cat}/K_m (1/s/M)	980 ± 60

Table S11. Kinetic parameters determined for GriG.

K_m (mM)	4.2 ± 0.8
V_{max} (μM/min)	0.80 ± 0.15
k_{cat} (1/s)	0.016 ± 0.002
k_{cat}/K_m (1/M/s)	4.0 ± 0.6

Table S12. Primers used for cosmid modification.

No.	sequence of primer
1	CGTGCCGGCGTACCACTCTCGTACGGGTTCGGGGAGGCGTCGACCGTGCTTACGGGGTCTGACGCTCAGTGG
2	*GCCGCCGCGTGCCGGACGTCTGTTCCGCCCTTCCCGGGGCCCCTGCGCGG***ACTAGT**TTAGAAAAACTCATCGAGCATC
3	*ACGTAACCGTCACTCTTGTACTTTTCGACCTGATCGGCCGTGAGCTGCAT***TCTAGA**TTACGCCCCGCCCTGCCACTC
4	*CCTGCAGCGCCGGTTCGATCTCACCAAAGGGCCGTCCATCCGCTTCACCT***ATTTAAAT**CCGGCGGTGCTTTTGCCGTTAC
5	*CGGCCGATCAGGTCGAAAAGTACAAGAGTGACGGTTACGTCCTTCTTGAA***CTTAAG**CCGGCGGTGCTTTTGCCGTT
6	*CTCGATTCGGCGGCCTCATGCAGCGGTCCGCTCCTGGAGACCAGAGGCGT***CTTAAG**TTACGCCCCGCCCTGCCACTC
7	*GGTCTCCGTCTGTGGTAACCCCTGGTGTGGCGTCCGGCGGCATCACTGCC***CTTAAG**CCGGCGGTGCTTTTGCCGTT
8	*ACCGTGGGAAGGTGCCGGCTTTCCAGTACTGCCTTCACCACCTCGCCGGT***CTTAAG**TTACGCCCCGCCCTGCCACT
9	*ATCACTTTGGAGGCAGAAATATGCCAATTGCCACGGTGAACGAGACCCAA***CTTAAG**CCGGCGGTGCTTTTGCCGTT
10	*GAGAAGAAGTCGAGTATGTGCTTGTTGACGACATCAGGTTGCTCCAGGTA***CTTAAG**TTACGCCCCGCCCTGCCACT
11	*GGATGCGCTATGGCGTCGTGATTCTCCCTGAACACAGCTGGGCCAGAGCC***CTTAAG**CCGGCGGTGCTTTTGCCGTT
12	*CCTGGTGCGGTCGGCAGGATACTTGCGATGTCCTCCATGACCGCGGGGTC***CTTAAG**TTACGCCCCGCCCTGCCACT

V. Supplementary References

1. T. Kieser, Bibb, M., Buttner, M.J., Chater, K.F., Hopwood, D.A., *Practical Streptomyces Genetics*, The John Innes Foundation, Norwich, England, 2000.
2. M. Bierman, R. Logan, K. O'Brien, E. T. Seno, R. N. Rao and B. E. Schoner, *Gene*, 1992, **116**, 43-49.
3. Z. Xu, K. Jakobi, K. Welzel and C. Hertweck, *Chemistry & biology*, 2005, **12**, 579-588.
4. R. D. Finn, A. Bateman, J. Clements, P. Coggill, R. Y. Eberhardt, S. R. Eddy, A. Heger, K. Hetherington, L. Holm, J. Mistry, E. L. Sonnhammer, J. Tate and M. Punta, *Nucleic Acids Res*, 2014, **42**, D222-230.
5. S. F. Altschul, W. Gish, W. Miller, E. W. Myers and D. J. Lipman, *Journal of molecular biology*, 1990, **215**, 403-410.
6. T. Weber, K. Blin, S. Duddela, D. Krug, H. U. Kim, R. Bruccoleri, S. Y. Lee, M. A. Fischbach, R. Muller, W. Wohlleben, R. Breitling, E. Takano and M. H. Medema, *Nucleic Acids Res*, 2015, **43**, W237-243.
7. B. O. Bachmann and J. Ravel, *Methods in enzymology*, 2009, **458**, 181-217.
8. A. Kling, P. Lukat, D. V. Almeida, A. Bauer, E. Fontaine, S. Sordello, N. Zaburannyi, J. Herrmann, S. C. Wenzel, C. Koenig, N. C. Ammerman, M. B. Barrio, K. Borchers, F. Bordon-Pallier, M. Broenstrup, G. Courtemanche, M. Gerlitz, M. Geslin, P. Hammann, D. W. Heinz, H. Hoffmann, S. Klieber, M. Kohlmann, M. Kurz, C. Lair, H. Matter, E. Nuermberger, S. Tyagi, L. Fraisse, J. H. Grosset, S. Lagrange and R. Mueller, *Science*, 2015, **348**, 1106-1112.
9. B. Gust, G. L. Challis, K. Fowler, T. Kieser and K. F. Chater, *Proceedings of the National Academy of Sciences of the United States of America*, 2003, **100**, 1541-1546.
10. J. P. Muyrers, Y. Zhang and A. F. Stewart, *Genetic engineering*, 2000, **22**, 77-98.
11. Y. Zhang, F. Buchholz, J. P. Muyrers and A. F. Stewart, *Nature genetics*, 1998, **20**, 123-128.
12. J. E. Becker, R. E. Moore and B. S. Moore, *Gene*, 2004, **325**, 35-42.
13. D. Hoffmann, J. M. Hevel, R. E. Moore and B. S. Moore, *Gene*, 2003, **311**, 171-180.
14. C. Rausch, T. Weber, O. Kohlbacher, W. Wohlleben and D. H. Huson, *Nucleic Acids Res*, 2005, **33**, 5799-5808.
15. T. Stachelhaus, H. D. Mootz and M. A. Marahiel, *Chemistry & biology*, 1999, **6**, 493-505.
16. G. L. Challis, J. Ravel and C. A. Townsend, *Chemistry & biology*, 2000, **7**, 211-224.
17. T. Stachelhaus and M. A. Marahiel, *J Biol Chem*, 1995, **270**, 6163-6169.
18. H. Luesch, D. Hoffmann, J. M. Hevel, J. E. Becker, T. Golakoti and R. E. Moore, *J Org Chem*, 2003, **68**, 83-91.
19. R. Bhushan and H. Bruckner, *Journal of chromatography. B, Analytical technologies in the biomedical and life sciences*, 2011, **879**, 3148-3161.
20. T. Siegl, B. Tokovenko, M. Myronovskyi and A. Luzhetskyy, *Metabolic engineering*, 2013, **19**, 98-106.
21. L. Palego, G. Giannaccini, G. Saccomanni, A. Rossi, V. Lucchesi, G. Mascia, L. Betti, C. Manera, L. Bazzichi and A. Lucacchini, *Chromatographia*, 2010, **71**, 291-297.
22. S. L. Fu and R. T. Dean, *The Biochemical journal*, 1997, **324 (Pt 1)**, 41-48.
23. K. Buntin, K. J. Weissman and R. Muller, *Chembiochem : a European journal of chemical biology*, 2010, **11**, 1137-1146.
24. D. Isabelle, D. R. Simpson and L. Daniels, *Applied and environmental microbiology*, 2002, **68**, 5750-5755.
25. F. W. Studier, *Protein Expr Purif*, 2005, **41**, 207-234.
26. E. Gasteiger, A. Gattiker, C. Hoogland, I. Ivanyi, R. D. Appel and A. Bairoch, *Nucleic Acids Res*, 2003, **31**, 3784-3788.
27. F. Gorrec, *Journal of applied crystallography*, 2009, **42**, 1035-1042.
28. C. Vonrhein, C. Flensburg, P. Keller, A. Sharff, O. Smart, W. Paciorek, T. Womack and G. Bricogne, *Acta crystallographica. Section D, Biological crystallography*, 2011, **67**, 293-302.
29. W. Kabsch, *Acta crystallographica. Section D, Biological crystallography*, 2010, **66**, 125-132.
30. P. Evans, *Acta crystallographica. Section D, Biological crystallography*, 2006, **62**, 72-82.
31. P. R. Evans and G. N. Murshudov, *Acta crystallographica. Section D, Biological crystallography*, 2013, **69**, 1204-1214.
32. P. A. Karplus and K. Diederichs, *Science*, 2012, **336**, 1030-1033.
33. A. J. McCoy, R. W. Grosse-Kunstleve, P. D. Adams, M. D. Winn, L. C. Storoni and R. J. Read, *Journal of applied crystallography*, 2007, **40**, 658-674.
34. P. D. Adams, P. V. Afonine, G. Bunkoczi, V. B. Chen, I. W. Davis, N. Echols, J. J. Headd, L. W. Hung, G. J. Kapral, R. W. Grosse-Kunstleve, A. J. McCoy, N. W. Moriarty, R. Oeffner, R. J. Read, D. C. Richardson, J. S. Richardson, T. C. Terwilliger and P. H. Zwart, *Acta crystallographica. Section D, Biological crystallography*, 2010, **66**, 213-221.
35. P. Emsley, B. Lohkamp, W. G. Scott and K. Cowtan, *Acta crystallographica. Section D, Biological crystallography*, 2010, **66**, 486-501.
36. P. V. Afonine, R. W. Grosse-Kunstleve, N. Echols, J. J. Headd, N. W. Moriarty, M. Mustyakimov, T. C. Terwilliger, A. Urzhumtsev, P. H. Zwart and P. D. Adams, *Acta crystallographica. Section D, Biological crystallography*, 2012, **68**, 352-367.
37. L. A. Kelley and M. J. Sternberg, *Nat Protoc*, 2009, **4**, 363-371.
38. R. Nyfeler and W. Keller-Schierlein, *Helv Chim Acta*, 1974, **57**, 2459-2477.
39. C. Keller-Juslén, M. Kuhn, H. R. Loosli, T. J. Petcher, H. P. Weber and A. von Wartburg, *Tetrahedron Letters*, 1976, **17**, 4147-4150.
40. R. Traber, C. Keller-Juslén, H.-R. Loosli, M. Kuhn and A. Von Wartburg, *Helv Chim Acta*, 1979, **62**, 1252-1267.
41. O. D. Hensens, J. M. Liesch, D. L. Zink, J. L. Smith, C. F. Wichmann and R. E. Schwartz, *The Journal of antibiotics*, 1992, **45**, 1875-1885.

42. C. H. Chen, G. Lang, M. I. Mitova, A. C. Murphy, A. L. Cole, L. B. Din, J. W. Blunt and M. H. Munro, *J Org Chem*, 2006, **71**, 7947-7951.
43. H. Mori, Y. Urano, T. Kinoshita, S. Yoshimura, S. Takase and M. Hino, *The Journal of antibiotics*, 2003, **56**, 181-185.
44. K. Fujii, K. Sivonen, K. Adachi, K. Noguchi, H. Sano, K. Hirayama, M. Suzuki and K.-i. Harada, *Tetrahedron Letters*, 1997, **38**, 5525-5528.
45. D. P. Fewer, J. Jokela, L. Rouhiainen, M. Wahlsten, K. Koskenniemi, L. J. Stal and K. Sivonen, *Molecular microbiology*, 2009, **73**, 924-937.
46. A. R. Anas, T. Kisugi, T. Umezawa, F. Matsuda, M. R. Campitelli, R. J. Quinn and T. Okino, *J Nat Prod*, 2012, **75**, 1546-1552.
47. L. Liu, J. Jokela, L. Herfindal, M. Wahlsten, J. Sinkkonen, P. Permi, D. P. Fewer, S. O. Doskeland and K. Sivonen, *ACS chemical biology*, 2014, **9**, 2646-2655.
48. T. Golakoti, W. Y. Yoshida, S. Chaganty and R. E. Moore, *J Nat Prod*, 2001, **64**, 54-59.
49. M. Hibi, T. Kawashima, P. M. Sokolov, S. V. Smirnov, T. Kodera, M. Sugiyama, S. Shimizu, K. Yokozeki and J. Ogawa, *Appl Microbiol Biotechnol*, 2013, **97**, 2467-2472.
50. W. Jiang, R. A. Cacho, G. Chiou, N. K. Garg, Y. Tang and C. T. Walsh, *Journal of the American Chemical Society*, 2013, **135**, 4457-4466.
51. I. Hofer, M. Crusemann, M. Radzom, B. Geers, D. Flachshaar, X. Cai, A. Zeeck and J. Piel, *Chemistry & biology*, 2011, **18**, 381-391.
52. A. Copeland, S. Lucas, A. Lapidus, K. Barry, T. Glavina del Rio, E. Dalin, H. Tice, S. Pitluck, B. Foster, J. Schmutz, F. Larimer, M. Land, L. Hauser, N. Kyrpides, N. Ivanova, P. R. Jensen, B. S. Moore, K. Penn, C. Jenkins, D. Udwary, L. Xiang, E. Gontang and P. Richardson, ed. U. D. J. G. Institute, 2014.
53. J. Bursy, A. J. Pierik, N. Pica and E. Bremer, *J Biol Chem*, 2007, **282**, 31147-31155.
54. R. A. Cacho, W. Jiang, Y. H. Chooi, C. T. Walsh and Y. Tang, *Journal of the American Chemical Society*, 2012, **134**, 16781-16790.
55. L. Chen, Q. Yue, X. Zhang, M. Xiang, C. Wang, S. Li, Y. Che, F. J. Ortiz-Lopez, G. F. Bills, X. Liu and Z. An, *BMC genomics*, 2013, **14**, 339.
56. S. Houwaart, L. Youssar and W. Huttel, *Chembiochem : a European journal of chemical biology*, 2014, **15**, 2365-2369.
57. H. Mori, T. Shibasaki, K. Yano and A. Ozaki, *Journal of bacteriology*, 1997, **179**, 5677-5683.
58. P. Petzsch, A. Poehlein, D. B. Johnson, R. Daniel, M. Schlomann and M. Muhling, *Genome announcements*, 2015, **3**.
59. K. C. Y. Lee, X. C. Morgan, P. F. Dunfield, I. Tamas, K. M. Houghton, M. Vyssotski, J. L. J. Ryan, K. Lagutin, I. R. McDonald and M. B. Stott, ed. G. S. Extremophile Research Laboratory, Private Bag 2000, Taupo, 3353, NEW ZEALAND, 2015.
60. Y. Wang, Y. Chen, Q. Shen and X. Yin, *Gene*, 2011, **483**, 11-21.
61. B. A. Gruning, A. Erxleben, A. Hahnlein and S. Gunther, *Genome announcements*, 2013, **1**.
62. C. Muller, S. Nolden, P. Gebhardt, E. Heinzelmann, C. Lange, O. Puk, K. Welzel, W. Wohlleben and D. Schwartz, *Antimicrobial agents and chemotherapy*, 2007, **51**, 1028-1037.
63. V. Miao, R. Brost, J. Chapple, K. She, M. F. Gal and R. H. Baltz, *Journal of industrial microbiology & biotechnology*, 2006, **33**, 129-140.
64. S. Rachid, L. Huo, J. Herrmann, M. Stadler, B. Kopcke, J. Bitzer and R. Muller, *Chembiochem : a European journal of chemical biology*, 2011, **12**, 922-931.
65. A. S. Eustaquio, R. P. McGlinchey, Y. Liu, C. Hazzard, L. L. Beer, G. Florova, M. M. Alhamadsheh, A. Lechner, A. J. Kale, Y. Kobayashi, K. A. Reynolds and B. S. Moore, *Proceedings of the National Academy of Sciences of the United States of America*, 2009, **106**, 12295-12300.
66. M. Koberska, J. Kopecky, J. Olsovska, M. Jelinkova, D. Ulanova, P. Man, M. Flieger and J. Janata, *Folia microbiologica*, 2008, **53**, 395-401.
67. W. Li, A. Khullar, S. Chou, A. Sacramo and B. Gerratana, *Applied and environmental microbiology*, 2009, **75**, 2869-2878.
68. W. Li, S. Chou, A. Khullar and B. Gerratana, *Applied and environmental microbiology*, 2009, **75**, 2958-2963.
69. A. H. Deutch, C. J. Smith, K. E. Rushlow and P. J. Kretschmer, *Nucleic Acids Res*, 1982, **10**, 7701-7714.
70. M. A. Larkin, G. Blackshields, N. P. Brown, R. Chenna, P. A. McGettigan, H. McWilliam, F. Valentin, I. M. Wallace, A. Wilm, R. Lopez, J. D. Thompson, T. J. Gibson and D. G. Higgins, *Bioinformatics*, 2007, **23**, 2947-2948.
71. F. Jeanmougin, J. D. Thompson, M. Gouy, D. G. Higgins and T. J. Gibson, *Trends in biochemical sciences*, 1998, **23**, 403-405.
72. E. Conti, T. Stachelhaus, M. A. Marahiel and P. Brick, *Embo J*, 1997, **16**, 4174-4183.
73. M. Rottig, M. H. Medema, K. Blin, T. Weber, C. Rausch and O. Kohlbacher, *Nucleic Acids Res*, 2011, **39**, W362-367.
74. K. Reuter, M. Pittelkow, J. Bursy, A. Heine, T. Craan and E. Bremer, *PLoS One*, 2010, **5**, e10647.
75. M. A. McDonough, K. L. Kavanagh, D. Butler, T. Searls, U. Oppermann and C. J. Schofield, *J Biol Chem*, 2005, **280**, 41101-41110.
76. L. C. Blasiak, F. H. Vaillancourt, C. T. Walsh and C. L. Drennan, *Nature*, 2006, **440**, 368-371.
77. C. Wong, D. G. Fujimori, C. T. Walsh and C. L. Drennan, *Journal of the American Chemical Society*, 2009, **131**, 4872-4879.
78. Y. Minowa, M. Araki and M. Kanehisa, *Journal of molecular biology*, 2007, **368**, 1500-1517.
